# Three-Dimensional Printing and Bioprinting Strategies for Cardiovascular Constructs: From Printing Inks to Vascularization

**DOI:** 10.3390/polym17172337

**Published:** 2025-08-28

**Authors:** Min Suk Kim, Yuri Choi, Keel Yong Lee

**Affiliations:** 1 Department of Integrative Bioscience and Biotechnology, Sejong University, Seoul 05006, Republic of Korea; 2 Institute of Bioscience and Biotechnology, Sejong University, Seoul 05006, Republic of Korea

**Keywords:** bioink, 3D printing, 3D bioprinting, cardiovascular tissue engineering, vascularization, melt electrowriting, two-photon polymerization (2PP)

## Abstract

Advancements in bioinks and three-dimensional (3D) printing and bioprinting have significantly advanced cardiovascular tissue engineering by enabling the fabrication of biomimetic cardiac and vascular constructs. Traditional 3D printing has contributed to the development of acellular scaffolds, vascular grafts, and patient-specific cardiovascular models that support surgical planning and biomedical applications. In contrast, 3D bioprinting has emerged as a transformative biofabrication technology that allows for the spatially controlled deposition of living cells and biomaterials to construct functional tissues in vitro. Bioinks—derived from natural biomaterials such as collagen and decellularized matrix, synthetic polymers such as polyethylene glycol (PEG) and polycaprolactone (PCL), or hybrid combinations—have been engineered to replicate extracellular environments while offering tunable mechanical properties. These formulations ensure biocompatibility, appropriate mechanical strength, and high printing fidelity, thereby maintaining cell viability, structural integrity, and precise architectural resolution in the printed constructs. Advanced bioprinting modalities, including extrusion-based bioprinting (such as the FRESH technique), droplet/inkjet bioprinting, digital light processing (DLP), two-photon polymerization (TPP), and melt electrowriting (MEW), enable the fabrication of complex cardiovascular structures such as vascular patches, ventricle-like heart pumps, and perfusable vascular networks, demonstrating the feasibility of constructing functional cardiac tissues in vitro. This review highlights the respective strengths of these technologies—for example, extrusion’s ability to print high-cell-density bioinks and MEW’s ultrafine fiber resolution—as well as their limitations, including shear-induced cell stress in extrusion and limited throughput in TPP. The integration of optimized bioink formulations with appropriate printing and bioprinting platforms has significantly enhanced the replication of native cardiac and vascular architectures, thereby advancing the functional maturation of engineered cardiovascular constructs.

## 1. Introduction

**Figure 1:**
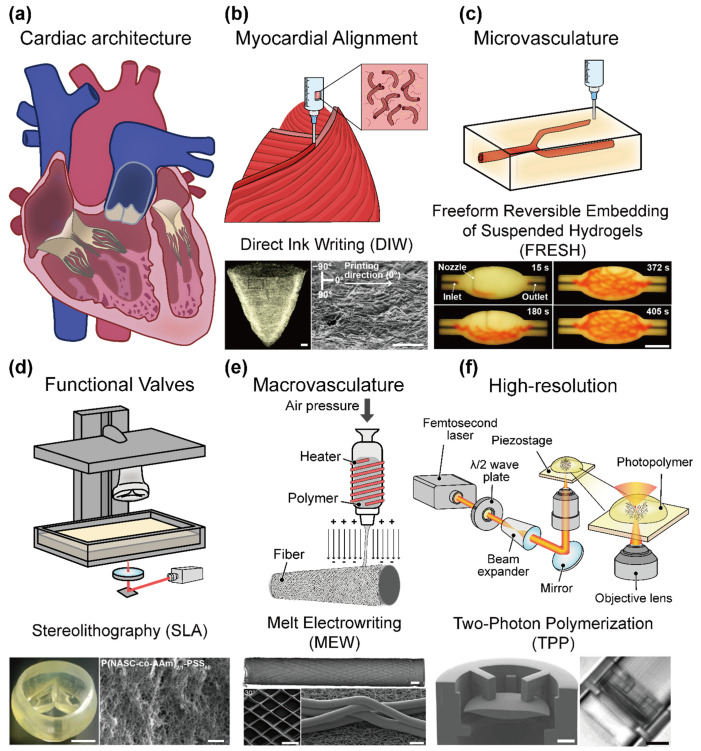
Overview of the current strategies and challenges in 3D bioprinting for cardiovascular tissue engineering. (**a**) Schematic illustration of the hierarchical structure of cardiac tissues. (**b**) Fiber-infused gel scaffolds guide cardiomyocyte alignment in 3D-printed ventricles [scale bar = 1 mm (**left**), 200 µm (**right**)]. Adapted with permission from Ref. [7], Springer Nature. (**c**) Biomanufacturing of organ-specific tissues with high cellular density and embedded vascular channels (scale bar = 10 mm). Adapted with permission from Ref. [12], The American Association for the Advancement of Science. (**d**) Biomechanically compatible hydrogel bioprosthetic valves [scale bar = 5 mm (**left**), 1 µm (**right**)]. Adapted with permission from Ref. [30], American Chemical Society. (**e**) Heterotypic scaffold design orchestrates primary cell organization and phenotypes in cocultured small-diameter vascular grafts [scale bar = 1 mm (**top**), 400 μm (**left-bottom**), 40 μm (**right-bottom**)]. Adapted with permission from Ref. [31], John Wiley and Sons. (**f**) Engineering a living cardiac pump on a chip using high-precision fabrication [scale bar = 50 µm (**left**), 100 µm (**right**)]. Adapted with permission from Ref. [32], The American Association for the Advancement of Science.

Cardiovascular diseases (CVDs) are the leading cause of mortality worldwide, accounting for approximately 17.9 million deaths annually [1]. This burden is exacerbated by the limited regenerative capacity of the adult myocardium and the structural complexity of the vascular networks, which restrict effective healing following injury [2,3]. Ischemic injury (e.g., myocardial infarction) can result in the loss of up to a billion cardiomyocytes, and owing to the limited regenerative capacity of the adult myocardium, these lost cells cannot be replaced, leading to fibrotic scar formation and compromised cardiac function [1]. Healing is further impeded by the heart’s intricate vascular network; the hierarchical architecture of large vessels down to capillaries is difficult to re-establish after injury, leaving regenerated muscle poorly perfused. Even when interventions, such as pharmacological therapy or surgical revascularization, are applied, they rarely restore the native tissue architecture or long-term physiological function of the heart. In the myocardium, structure and function are tightly coupled; the ventricular muscle fibers are aligned in specific orientations to maximize contractility, and the thick cardiac walls demand a dense coronary vasculature to deliver oxygen and nutrients [2,3,4] (Figure 1a). This critical interplay between form and function underscores the need for engineered platforms that can mimic native cardiac tissue structures to recapitulate its function. Three-dimensional (3D) printing and bioprinting technologies have both significantly contributed to cardiovascular tissue engineering. Conventional 3D printing, although acellular, has played a crucial role in creating patient-specific anatomical models, vascular grafts, and structural scaffolds that aid in pre-surgical planning, device testing, and the development of biomedical implants [5,6,7] (Figure 1b). These applications demonstrate how the ability to fabricate precise cardiovascular geometries has already benefited the biomedical and clinical communities by enabling more accurate simulation of complex cardiac structures and surgical interventions.

On the other hand, 3D bioprinting has emerged as a transformative biofabrication technology that offers spatially controlled deposition of living cells and biomaterials to construct anatomically precise, functional cardiovascular tissues [5,6,7] (Figure 1b). Unlike traditional tissue engineering methods, 3D bioprinting allows for the creation of complex, multicellular architectures guided by imaging data and computer-aided design. This approach holds particular promise for addressing the dual challenges of replicating myocardial contractility and generating perfusable vascular networks, both of which are essential for sustained tissue viability and function [6,8].

Recent advances have centered on the development of sophisticated bioinks—hydrogel-based formulations that encapsulate cells while mimicking the mechanical and biochemical milieu of native cardiac and vascular tissues. For example, hybrid bioinks that combine natural extracellular matrix components with synthetic polymers offer tunable stiffness and rheological properties, thereby improving print fidelity and cell viability. Such materials enable the fabrication of anisotropic cardiac constructs (with aligned cardiomyocytes for proper contraction) and endothelialized microvessels within the printed tissue [9,10,11]. Notably, new biofabrication methods, such as freeform reversible embedding of suspended hydrogels (FRESH), have achieved microscale resolution (~20 μm) in printing collagen-based scaffolds, yielding constructs with sufficient mechanical integrity for perfusion and seeding with vascular cells [9,12] (Figure 1c). These improvements in bioink design and printing techniques have greatly enhanced the cytocompatibility and maintenance of tissue-specific functions in vitro. Despite this progress, a fundamental hurdle in engineering thick functional tissues is the diffusion limits of oxygen and nutrients. In the absence of blood perfusion, simple diffusion can only support cells within a distance of 100–200 μm from a nutrient source [12,13,14]. Constructs larger than a few hundred microns quickly develop necrotic cores if they are not vascularized, reflecting the same limit observed in vivo in cell-dense tissues. Overcoming this barrier requires the incorporation of perfusable vasculature that can sustain cell survival throughout the tissue volume.

A number of bioprinting strategies have been explored to incorporate vascular networks into engineered cardiac tissues. In sacrificial ink printing approaches, a fugitive material (e.g., carbohydrate glass or pluronic hydrogel [15,16]) is printed in the shape of a vascular tree and later dissolved, leaving open channels that can be lined with endothelium [17]. Similarly, coaxial extrusion bioprinting uses concentric nozzles to directly print tubular structures with an inner core (often a sacrificial gel or support medium) and an outer cell-laden shell, effectively bioprinting blood vessel analogs in a single step [4]. Embedded printing in support baths (such as the FRESH method) has also enabled the creation of complex branching vessels by printing bioink within a temporary gel support that maintains the geometry until it is cured [17,18,19,20].

These methods have successfully produced multiscale vascular channels; however, challenges remain in achieving true capillary-level resolution, scaling up to clinically relevant tissue sizes, and maintaining long-term perfusion and remodeling of the printed vessels. Even though the most advanced extrusion bioprinters typically print channels on the order of hundreds of microns in diameter, dense capillary networks (<100 μm) require further improvements in printing resolution or the incorporation of self-assembling microvasculature [21]. Ensuring that printed vessels can support physiological flow without thrombosis or leakage (and that they can integrate with host vasculature in vivo) is an additional issue that is the focus of ongoing research [22,23,24].

In this context, there is a growing interest in microfluidic-assisted bioprinting and dynamic culture strategies to enhance vascularization. By integrating microfluidic channels or perfusion systems into the bioprinting workflow, researchers can introduce flow immediately after or even during the printing process, providing endothelial cells with shear stress cues necessary for maturation. Microfluidic perfusion of bioprinted vascular channels not only supplies nutrients and oxygen deeper into the tissue but also guides endothelial cell alignment in the direction of flow, promoting the formation of a stable endothelium that closely resembles native blood vessels. Studies have shown that applying flow through printed channels leads to improved endothelial barrier function (e.g., selective permeability and anti-thrombogenic quiescence) and even induces self-remodeling of nascent vessels [25,26]. For example, coaxial extrusion was used to develop a vascular network that allowed perfusion, where shear stress facilitated the formation of cell–cell junctions and promoted angiogenic sprouting in response to growth factors [27].

The convergence of 3D printing, 3D bioprinting, and microfluidic systems has paved the way for the development of cardiovascular organ-on-a-chip platforms. By coupling bioprinted cardiac tissues (e.g., patches of myocardial fibers with embedded vessels) with microfluidic circuitry, researchers can create microphysiological systems that recapitulate the hemodynamic environment of the human heart and vasculature. These systems allow for the controllable and reproducible simulation of blood flow, pressure, and multi-organ interactions, providing more predictive models for disease research and drug screening than conventional 2D cell cultures [28,29]. Human-mimetic cardiovascular chips serve as an intermediate between simplistic in vitro assays and complex in vivo models, embodying patient-specific anatomy and physiology.

As printing and bioprinting technologies continue to advance, the fabrication of clinically relevant and fully vascularized cardiac tissues is becoming an increasingly tangible goal. Improvements in printer resolution and fidelity now enable the recreation of previously unattainable cardiac microstructures (such as capillary networks and aligned myocardial laminae) (Figure 1d) [30]. The integration of advanced imaging techniques with printing workflows further enhances the precision and personalization of engineered tissues (Figure 1e,f) [31,32]. High-resolution magnetic resonance imaging (MRI) and computed tomography (CT) can be used to derive patient-specific anatomical models, which in turn guide printers and bioprinters to produce tissue constructs tailored to an individual’s geometry and pathology. Notably, a recent study demonstrated the feasibility of 3D printing and bioprinting a small human heart model from collagen bioink using MRI/CT data, with the printed heart replicating the patient’s anatomical structure when evaluated via micro-CT imaging.

Furthermore, the incorporation of smart materials and stimuli-responsive bioinks has opened new avenues for creating dynamic cardiovascular constructs. Hydrogels that respond to stimuli (e.g., pH, temperature, electrical, or mechanical cues) can be used to fabricate 4D-bioprinted tissues that change their properties over time or in reaction to external triggers. For example, stimuli-responsive biomaterials have been employed to deliver drugs or growth factors in a controlled fashion within cardiac patches and to adjust scaffold stiffness or ligand presentation in response to mechanical loading, thereby mimicking adaptive remodeling of the heart [33]. In summary, the synergy among 3D printing, 3D bioprinting, microfluidic perfusion, and advanced biomaterials has driven the development of human-mimetic cardiovascular platforms. These bioengineered systems strive to recapitulate the critical structure–function relationships of the heart and vasculature, bringing us closer to viable solutions for cardiac repair, disease modeling, and drug development in the fight against cardiovascular disease (Figure 1).

## 2. Bioink Development for Cardiovascular Tissue Engineering

Bioinks constitute the cornerstone of 3D bioprinting technology in cardiovascular tissue engineering, directly influencing not only the precision and structural fidelity of printed constructs but also their long-term cell viability, tissue maturation, and functional performance [10,34]. An optimal bioink formulation for cardiovascular applications should exhibit the following characteristics: (i) high biocompatibility to support cell survival and proliferation, (ii) appropriate rheological properties that ensure printability and shape fidelity, (iii) tunable mechanical strength mimicking the physiological stiffness and elasticity of native cardiac and vascular tissues, and (iv) bioactivity to facilitate endothelialization, angiogenesis, and hierarchical vascular network formation within engineered tissues.

To date, diverse bioink formulations have been developed and categorized into three primary groups: natural bioinks derived from extracellular matrix components and biopolymers; synthetic bioinks fabricated from synthetic polymers; and hybrid bioinks that combine the advantageous properties of both natural and synthetic materials. Each bioink category presents unique benefits and challenges, making careful selection and optimization essential for specific cardiovascular tissue engineering applications. The following sections provide a comprehensive evaluation of recent developments, advantages, limitations, and applications of these bioink categories in cardiovascular bioprinting:

### 2.1. Natural Bioinks

**Figure 2:**
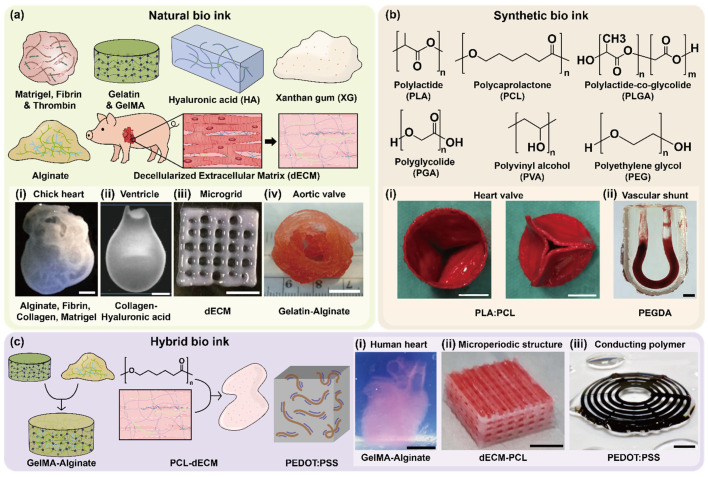
Classification of bioinks for cardiovascular bioprinting based on their origin and composition. (**a**(**i**)) Three-dimensional printing of complex biological structures by freeform reversible embedding of suspended hydrogels (scale bar = 1 cm). Adapted with permission from Ref. [19], The American Association for the Advancement of Science. (**a**(**ii**)) Direct 3D bioprinting of hiPSC-derived cardiomyocytes to generate functional cardiac tissues (scale bar = 2 mm). Adapted with permission from Ref. [36], John Wiley and Sons. (**a**(**iii**)) Printing three-dimensional tissue analogs with decellularized extracellular matrix bioink (scale bar = 5 mm). Adapted with permission from Ref. [38], Springer Nature. (**a**(**iv**)) 3D bioprinting of heterogeneous aortic valve conduits with alginate/gelatin hydrogels (scale bar = 1 cm). Adapted with permission from Ref. [67], John Wiley and Sons. (**b**(**i**)) On-demand heart valve manufacturing using focused rotary jet spinning (scale bar = 10 mm). Adapted with permission from Ref. [48], Elsevier. (**b**(**ii**)) Blood flow within bioengineered 3D-printed vascular constructs using the porcine model (scale bar = 2 mm). Adapted with permission from Ref. [51], Frontiers Media S.A. (**c**(**i**)) Cation-crosslinked κ-carrageenan sub-microgel medium for high-quality embedded bioprinting (scale bar = 1 cm). Adapted with permission from Ref. [53], IOP Publishing Ltd. (**c**(**ii**)) Printing three-dimensional tissue analogues with decellularized extracellular matrix–PCL composite bioink (scale bar = 5 mm). Adapted with permission from Ref. [38], Springer Nature. (**c**(**iii**)) 3D printing of conducting polymers (scale bar = 2 mm). Adapted with permission from Ref. [57], Springer Nature.

Natural bioinks, primarily derived from extracellular matrix (ECM) components or biopolymers, are highly favored for cardiovascular bioprinting because of their inherent biocompatibility, biodegradability, and bioactivity. These bioinks facilitate cellular interactions, support tissue-specific differentiation, and promote vascularization, thus playing a crucial role in recreating the native microenvironment of cardiovascular tissues (Figure 2a).

Fibrin, a rapidly gelling and naturally occurring protein, actively promotes angiogenesis, endothelial adhesion, and early-stage capillary formation (Figure 2a(i)) [19]. Its intrinsic biological activity accelerates the vascularization process in engineered tissues; however, fibrin-based bioinks often suffer from rapid degradation [35], limiting their long-term mechanical stability and functional performance. Thus, fibrin bioinks are commonly combined with mechanically robust materials or crosslinkers to enhance the structural longevity of the cardiovascular constructs.

Collagen, the predominant structural protein in the native ECM, significantly enhances cellular adhesion, migration, and vascular remodeling (Figure 2a(ii)) [36]. While collagen bioinks effectively support tissue maturation and neovascularization, their limited mechanical strength [37] necessitates reinforcement through crosslinking strategies or the incorporation of synthetic polymer networks to achieve physiologically relevant tensile properties.

Decellularized extracellular matrix (dECM) bioinks, derived from native cardiovascular tissues, maintain complex biochemical and structural cues inherent to the original tissues (Figure 2a(iii)) [38]. Cardiac dECM bioinks specifically promote cardiomyocyte survival, differentiation, and functional maturation while also stimulating neovascularization within printed constructs [39,40]. Notably, cardiac-specific dECM bioinks have successfully supported the formation of vascularized cardiac patches and have been integrated into microfluidic organ-on-a-chip platforms [41], thereby effectively modeling the native complexity and function of cardiovascular tissue.

Gelatin, obtained through the partial hydrolysis of collagen, retains bioactive motifs such as integrin-binding arginine-glycine-aspartic acid (RGD) sequences, thereby supporting robust cell adhesion, spreading, and proliferation [42]. Gelatin exhibits temperature-sensitive gelation properties and is easy to handle. Its chemically modified variant, gelatin methacryloyl (GelMA) [43,44], can be photocrosslinked to yield constructs with tunable mechanical stiffness and enhanced structural integrity. Consequently, GelMA-based bioinks are extensively employed for fabricating anisotropic cardiac scaffolds and endothelialized microvascular networks, effectively replicating the native myocardial tissue structure and function (Figure 2a(iv )) [45].

Alginate, an anionic polysaccharide derived from brown algae, rapidly forms hydrogels through ionic crosslinking with divalent cations (e.g., Ca^2+^). Although alginate is inherently bioinert and lacks intrinsic cell-binding motifs, it offers excellent printability and structural stability [46]. To address its limited biological activity, alginate is typically combined with cell-adhesive molecules such as gelatin or GelMA to form hybrid bioinks that significantly enhance cytocompatibility. Alginate-based blends are particularly advantageous for coaxial extrusion bioprinting, enabling the direct fabrication of stable perfusable vascular channels and tubular constructs.

Natural bioinks provide essential biological cues and a physiologically relevant microenvironment, thus significantly advancing the fabrication of functional cardiovascular tissues. However, their mechanical limitations necessitate strategic combinations with synthetic or hybrid bioinks to meet the structural and functional criteria required for clinical translation.

### 2.2. Synthetic Bioinks

Synthetic bioinks are engineered polymeric materials that provide exceptional control over physicochemical characteristics, including mechanical stiffness, degradation kinetics, rheological behavior, and print fidelity. Despite their versatility and superior mechanical tunability, synthetic bioinks generally lack intrinsic biological functionality, necessitating functionalization with bioactive molecules or blending with natural bioinks to improve their cytocompatibility and tissue integration (Figure 2b).

Polycaprolactone (PCL) [47], a biodegradable thermoplastic polymer, is extensively employed in melt electrowriting (MEW) owing to its superior thermal stability, mechanical durability, and ability to be processed into microscale anisotropic fiber scaffolds. Similarly, polylactic acid (PLA) is utilized in MEW because of its advantageous printability and biodegradability. However, its relatively higher stiffness and reduced flexibility compared to PCL may constrain its applicability in certain soft tissue applications (Figure 2b(i,ii)) [48]. PCL fibers serve as robust mechanical frameworks for cardiovascular constructs, offering essential reinforcement, structural integrity, and directional guidance for cellular alignment and maturation. Although PCL is inherently bioinert, recent studies have shown enhanced bioactivity and cell interactions through the functionalization of PCL scaffolds with ECM-derived peptides, growth factors, or by coating with bioactive hydrogels.

Polyethylene glycol diacrylate (PEGDA) is widely employed in cardiovascular bioprinting because of its photopolymerization capability, highly tunable mechanical properties, and controlled degradation profiles. PEGDA-based inks have been directly applied for bioprinting implantable vascular constructs, where stereolithographically printed hydrogels were surgically connected to host vasculature and sustained physiological blood flow under arterial pressure (Figure 2b(ii)) [49]. PEGDA-based bioinks can be precisely tailored to mimic various mechanical environments, ranging from soft vascular tissues to stiffer cardiac structures. However, owing to PEGDA’s bioinert nature, PEGDA is commonly combined with bioactive components such as gelatin or dECM to enhance cell adhesion, viability, and tissue-specific functions [50,51].

Moreover, emerging synthetic bioinks based on shape-memory polymers (SMPs) and advanced copolymers, such as PCL-PEG-PCL triblock copolymers, have shown promise in addressing the mechanical and functional challenges of cardiovascular tissues [52]. These advanced polymers provide enhanced elasticity, flexibility, and dynamic responsiveness to physiological stimuli, thereby facilitating improved biomechanical compatibility and adaptive tissue remodeling after implantation.

Overall, synthetic bioinks offer significant potential for the precise engineering of cardiovascular tissues by providing mechanically tunable and structurally stable scaffolds. Ongoing research efforts continue to focus on integrating these synthetic materials with biologically active components to achieve both mechanical robustness and biological functionality, which are essential for the successful clinical translation of engineered cardiovascular constructs.

### 2.3. Hybrid Bioinks

Hybrid bioinks, which integrate natural and synthetic components, have been developed to synergistically combine bioactivity with mechanical robustness, making them particularly advantageous for engineering complex vascularized tissues that exhibit hierarchical structures (Figure 2c).

Alginate-GelMA and GelMA-HAMA (hyaluronic acid methacrylate) blends are commonly utilized in coaxial extrusion bioprinting to create perfusable microchannels. Alginate contributes to rapid ionic crosslinking, offering immediate structural stability, whereas GelMA and hyaluronic acid methacrylate (HAMA) provide adjustable mechanical properties, enhanced cellular adhesion, and bioactivity. These hybrid bioinks effectively support the endothelial cell lining, promoting the formation of functional, vascularized structures and microvessels that are essential for tissue viability and maturation (Figure 2c(i)) [46,53].

Nanoparticle-enhanced hybrid bioinks, which integrate materials such as gold nanorods or carbon nanotubes into GelMA-based formulations, have been extensively investigated. These nanoparticles not only substantially augment mechanical strength and structural fidelity but also enhance electrical conductivity and signal propagation, which are critical attributes for cardiac tissue constructs that necessitate synchronized cellular contraction [54]. Similarly, hybrid systems that incorporate polymers, such as polyethylene glycol (PEG)-fibrinogen and polyvinyl alcohol (PVA)-alginate composites, have been developed to achieve the spatial compartmentalization of distinct cell types within printed constructs. These hybrid bioinks enable the fabrication of complex structures such as Janus fibers or multilayered tissues, facilitating spatially controlled differentiation, vascularization, and functional heterogeneity [55]. To improve the mechanical stability of soft dECM hydrogels, hybrid constructs combining polycaprolactone (PCL) frameworks with tissue-specific dECM bioinks were devised. PCL microfibers provide structural support, whereas cell-laden dECM pre-gels occupy the interstices to maintain a bioactive microenvironment. A wider PCL spacing (200 μm) was employed for load-bearing cartilage tissue, and finer spacing (100 μm) was used for softer adipose tissue. Despite the stiffness of PCL, most cells remained within the compliant dECM, preserving high viability and supporting tissue-specific differentiation (Figure 2c(ii)) [38].

Recently, conductive hybrid bioinks, such as poly (3,4-ethylenedioxythiophene): polystyrene sulfonate (PEDOT:PSS)-enhanced GelMA bioinks, have emerged, which have significantly improved electrical conductivity, cardiomyocyte coupling, and overall electromechanical integration in engineered cardiac tissues (Figure 2c(iii)) [56,57]. Moreover, the application of advanced microfluidic-based bioprinting strategies, such as coaxial or flow-focusing methods, in hybrid bioinks has enabled the precise spatial patterning of endothelial cells alongside parenchymal cells within a single printed structure. This capability has allowed for the development of multicellular fibers and vascularized tissues that closely mimic the native vascular architecture, ensuring efficient nutrient delivery and metabolic waste removal through thick bioprinted constructs [20,58].

Thus, hybrid bioinks are uniquely positioned to overcome the limitations of natural or synthetic bioinks by delivering both the biological signaling and structural integrity required for successful cardiovascular tissue engineering. Continued advancement in hybrid bioink formulations and printing methodologies is expected to drive significant progress toward clinically relevant cardiovascular tissue constructs.

### 2.4. Functional Categorization of Bioinks

Bioinks used in cardiovascular tissue engineering can be functionally classified according to their primary roles, including vascularization, structural support, cellular guidance, and multicellular compartmentalization. The effective integration of bioinks across these categories is essential for closely recapitulating the structural complexity and functional heterogeneity of native cardiovascular tissues.

Vascularization-supporting bioinks primarily include fibrin, collagen, gelatin methacryloyl (GelMA), decellularized extracellular matrix (dECM), and alginate-based hybrid formulations. These bioinks facilitate endothelial cell adhesion, proliferation, and migration, thereby actively promoting angiogenic sprouting and formation of perfusable vascular networks. Their intrinsic bioactivity and ECM-like properties are critical for initiating early-stage neovascularization and sustaining long-term tissue viability through efficient nutrient and oxygen transport [35,37].

Structurally supportive bioinks, such as polycaprolactone (PCL) and polyethylene glycol diacrylate (PEGDA), as well as other mechanically robust synthetic polymers, provide the mechanical strength and elasticity required for cardiovascular applications. These materials can withstand dynamic mechanical stresses, including cyclic contractions of cardiac tissues and pulsatile shear forces in vascular environments. Their tunable stiffness, durability, and mechanical integrity ensure the structural stability and biomechanical compatibility of engineered cardiovascular constructs [50,59].

Cellular orientation-guiding bioinks encompass materials such as melt electrowriting (MEW)-aligned PCL fibers and micropatterned GelMA constructs. These bioinks facilitate anisotropic structural organization, which is crucial for the proper alignment and functional maturation of cardiomyocytes. The resulting aligned cellular architectures effectively replicated the native myocardial structure, enhanced synchronous contraction, and improved overall cardiac tissue functionality [45].

In addition, bioinks specifically designed for multicellular compartmentalization are instrumental in constructing spatially organized, complex tissue structures. These include bioinks suitable for microfluidic and coaxial extrusion techniques, enabling the formation of Janus fibers or multilayered tubular constructs. Such bioinks allow for the precise spatial arrangement of endothelial and parenchymal cells, promoting controlled cellular interactions, tissue complexity, and functional heterogeneity. This capability is essential for accurately modeling the intricate multicellular architecture observed in native cardiovascular tissues [55]. Function-based categorization highlights the need for the strategic integration of bioinks within engineered constructs, which combines vascularization capacity, mechanical robustness, cellular guidance, and spatial compartmentalization. This integrative approach significantly enhances the biological fidelity, functionality, and potential clinical relevance of bioprinted cardiovascular tissues.

### 2.5. Emerging and Stimuli-Responsive Bioinks

Recent advancements in bioink formulations have led to the development of dynamic and stimuli-responsive (often termed “4D-responsive”) bioinks, which are characterized by their ability to adapt their physical and biochemical properties over time or in response to specific environmental triggers. Such bioinks include shape-memory hydrogels, photoresponsive materials, and enzyme-degradable matrices, all designed to support time-dependent structural remodeling, controlled release of bioactive factors, and temporally staged vascularization processes essential for cardiovascular tissue maturation [60].

For instance, shape-memory hydrogels can undergo programmed structural changes triggered by temperature or other physiological cues, thereby facilitating gradual vascular integration or mechanical adaptation after implantation. Photo-responsive bioinks enable the precise spatiotemporal control of material crosslinking and degradation, allowing for the localized adjustment of tissue stiffness or the delivery of growth factors at specific developmental stages. Enzyme-degradable matrices are engineered to degrade gradually in response to enzymatic activities associated with tissue remodeling, thereby promoting the timely regeneration of native tissue architecture and integration with host tissues [60].

Additionally, conductive bioinks enriched with electrically active materials, such as graphene, poly(3,4-ethylenedioxythiophene): polystyrene sulfonate (PEDOT:PSS), and carbon nanotubes [61] (CNTs), have gained considerable attention because of their ability to improve electrical signal propagation and cardiomyocyte coupling. These conductive bioinks enhance synchronous contraction, promote physiological-level electrical conductivity, and improve the overall electromechanical integration in cardiac patches, thus addressing critical functional requirements for engineered myocardial tissues [45,54].

In parallel, bioink composition and biomaterial choices must also comply with regulatory frameworks established by the U.S. Food and Drug Administration (FDA) and the European Medicines Agency (EMA). Cardiovascular implants require validated biocompatibility, biodegradability, sterility, and scalable manufacturing processes, underscoring that translational success depends on both biological performance and regulatory approval. Furthermore, it is important to acknowledge that the majority of bioinks are currently in the research-grade hydrogel phase. Their successful translation necessitates overcoming significant challenges related to GMP-compliant manufacturing, reproducibility, and sterilization. Specifically, for cardiovascular applications, comprehensive regulatory testing in accordance with ISO 10993 [62] guidelines, including assessments of hemocompatibility and thrombogenicity, will be essential to ensure clinical safety and efficacy.

These emerging trends emphasize the need for continuous innovation in bioink technologies. The development of advanced bioinks, particularly those that integrate stimuli-responsive behavior and enhanced electrical conductivity, represents a significant step toward the fabrication of more physiologically relevant, clinically translatable cardiovascular constructs with dynamic functional capabilities.

### 2.6. AI-Assisted Bioink Optimization and Predictive Design

Artificial intelligence (AI) and machine learning (ML) are increasingly applied to accelerate bioink development and process optimization. Traditional trial-and-error approaches to balance rheological behavior, print fidelity, and biocompatibility are time-consuming and limited in scope. In contrast, AI-driven models can learn from experimental datasets to predict printability, mechanical stability, and cell viability, enabling the rational design of bioinks tailored for cardiovascular applications [63,64].

Deep learning and regression algorithms have been used to forecast extrusion stability, shear-induced cell damage, and optimal printing parameters, such as pressure and temperature. Generative models extend this capability by suggesting novel polymer combinations beyond those currently tested experimentally. Furthermore, AI integration allows for predictive modeling of post-printing cellular behavior, guiding strategies that anticipate tissue maturation and long-term function [65].

Overall, AI-assisted optimization represents a shift toward data-driven, predictive design of bioinks, reducing variability and accelerating translational progress. However, successful implementation will require standardized datasets, robust experimental validation, and clear regulatory pathways to ensure clinical reliability [66].

**Table 1:** Representative bioink formulations for 3D bioprinting of cardiovascular tissues.

Bioink Composition	Key Properties	Bioprinting Method	Crosslinking	Applications	Cited Models/Cell Type	References
Alginate, Methacrylated collagen, CNT	Electrically conductive, enhanced adhesion	Extrusion	Photo/ionic crosslinking	Cardiac patch development	HCAECs	[61]
Gelatin	Contact guidance, enhanced adhesion	Extrusion	Enzymatic	Cardiac patch development, in vitro cardiac modeling	Neonatal rat CMs, hMSCs	[42]
GelMa, HAGM, PEGDA	Contact guidance, Respond to inotropic agents at low concentrations	DLP	Photocrosslinking	in vitro cardiac modeling	Neonatal rat CMs	[44]
Cardiac dECM, GelMA, Eosin Y	Biochemical specificity, pro-angiogenic	Extrusion	Photocrosslinking	Cardiac patch development	hCPCs, rat CFBs	[40]
Gelatin, Fibronectin, HA, Fibrinogen, Thrombin	High cell viability, electrophysiological activity	Extrusion	Photocrosslinking	in vitro cardiac modeling	hiPSC-CMs, hCFBs	[68]
GelMA, Alginate	Contact guidance, bioactive, structural stability	Extrusion	Photo/ionic crosslinking	Endothelialized cardiac chips	Neonatal rat CMs, HUVECs	[18]
PCL, CNT	Electroconductivity, contact guidance	Extrusion	Thermal	in vitro cardiac modeling	H9c2	[47]
PCL, Human dECM	Regenerative, supports migration	Extrusion	Thermal	Epicardial stem cell patch	hCPCs, hMSCs	[39]
Alginate, PEGDA, Fibrinogen, Irgacure 295	Vascular integration, endothelialization	Extrusion	Photocrosslinking	Vascularized cardiac tissue engineering	iPSC-CMs, HUVECs	[49]
Carbopol, Alginate, Gelatin	Various complex structures were fabricated using ionic, photocrosslinkable and thermoresponsive bioinks by adjusting the concentration and pH of the Carbopol support bath	FRESH	Ionic crosslinking	Vascular tissue engineering	NIH 3T3	[69]
Adamantane/β-cyclodextrin-modified MeHA	Supramolecular assembly, guest–host interaction, free-standing structure fabrication	FRESH	Photocrosslinking	Spiral and branching channels	hMSCs, NIH 3T3	[70]
Pluronic F127 diacrylate, Pluronic F127	Microvascular structuring, tissue engineering, organ modeling	FRESH	Photocrosslinking	Microvascular networks	-	[15]
Pluronic F127, Alginate, GelMA	Continuous multimaterial extrusion, digitally tunable printhead, shear-thinning bioinks	FRESH	Photo/ionic crosslinking	Multimaterial tissue fabrication	HDFs, HepG2, hMSCs, HUVECs, MC3T3	[16]
Agarose, GelMA, Matrigel^®^, Fibronectin, Alginate	CLASS (agarose slurry support), freestanding construct stability, multi-bioink compatibility	FRESH	Photo/ionic crosslinking	Freestanding soft tissue printing, long-term in vitro culture	HEK 293	[43]
Perfluoro-tributylamine support bath, Agarose	Long-term structural stability, Cell viability, and proliferation	FRESH	Thermal	3D construct mimicking a vascular bifurcation and printed cylinders	hMSC, MG63	[71]
Gellan gum, Laponite, Alginate, Gelatin, PEGDA	Gellan support baths support the printing of functional bioinks that can crosslink with physical, enzymatic, and photocrosslinking mechanisms	FRESH	Ionic, enzymatic, photocrosslinking	Freestanding soft tissue printing	NIH 3T3	[72]
Alginate, Xanthan gum, Collagen, Carboxymethylcellulose	Biocompatibility, multi-cellular printing	FRESH	Thermal	Freestanding soft tissue printing, long-term in vitro culture	NIH 3T3, HUVECs	[73]
Collagen, Matrigel^®^, Gelatin (sacrificial)	High cell density, Perfusable vascular channels	FRESH	Thermal, enzymatic crosslinking	Organ-specific tissues, vascularized cardiac engineering, and pharmaceutical testing	HUVEC, iPSC-derived cardiac/cerebral organ building blocks	[12]
Gelatin, Pluronic F127, Gum arabic, Alginate, Collagen, Fibronectin, MeHA, Fibrinogen	Freeform constructs composed of multiple materials and nonplanar features	FRESH	Ionic, enzymatic crosslinking, pH-driven gelation	Freestanding soft tissue printing, vascularized cardiac tissue engineering	hESC-CMs, C2C12, MC3T3	[9,19,74,75]
PCL	Faster pore bridging for the radial pattern	MEW	Thermal	Heart valve tissue engineering, integration with cell-laden hydrogels	hUVSMCs	[76]
PCL	Contact guidance	MEW	Thermal	Scaffold design for orthopedic surgery, tubular and gradient scaffold design	MC3T3-E1	[77]
pHMGCL/PCL	Contact guidance	MEW	Thermal	Cardiac tissue engineering	CPCs	[78]
PCL	Contact guidance	MEW	Thermal	Anatomical model fabrication, long-term cell-laden structure maintenance	hMSCs	[79]
PCL	Elongation along the fibers for a higher laydown angle, while the lamellar shape of cells is on the smaller laydown angle	MEW	Thermal	Optimization of scaffold architecture for controlled cell confinement using machine learning	NHDFs	[80]
PCL	Increased cell proliferation and cell–cell interactions on more dense coils	MEW	Thermal	In vivo implantation scaffold, mechanically tunable scaffold platform	hMSCs	[81]
PCL	Unidirectional cell alignment for rhombus pores with increased gene expressions.	MEW	Thermal	in vitro renal tubule model, drug screening, regenerative medicine	ciPTECs, HUVECs	[82]
GelMA, GM-HA	μCOB bioprinting, prevascularized architecture, multi-cell encapsulation	DLP	Photocrosslinking	Engineering of vascularized tissue constructs with complex microarchitectures	HUVECs, C3H/10T1/2, SCID mice	[83]
GelMA, GM-HA	Hexagonal microarchitecture, triculture (hiPSC-HPCs, HUVECs	DLP	Photocrosslinking	3D triculture liver model	hiPSC-HPCs, ASCs, HUVECs	[84]
GelMA, GM-HA	Tri-regional patterning, HA-based, stiffness-tunable	DLP	Photocrosslinking	Tri-regional GBM model	GBM cells, HUVECs	[85]
GelMA, GM-HA	HA-rich hydrogel, multicellular co-culture (GSCs, astrocytes	DLP	Photocrosslinking	GBM environment model	GSCs, macrophages, NPCs, astrocytes	[86]
GelMA, dECM (Liver)	Tunable stiffness, UV curable	DLP	Photocrosslinking	Liver model of hepatocellular carcinoma	HepG2	[16]
GelMA, nHA	GelMA-nHA Composite supports stromal-cancer cell co-culture, stereolithographic printing	SLA	Photocrosslinking	Breast cancer model	MSCs, human osteoblasts, BrCa cells	[87]
OMA-PEGDA	Spatial co-culture, maskless patterning, tunable mechanical and degradation properties	SLA	Photocrosslinking	Co-culture of neurons and muscle myoblasts	Primary hippocampus neurons, skeletal muscle myoblasts	[88]
TAZ, DAS	Water-soluble, photocurable, high cell viability	2PP	Photocrosslinking	3D cell-laden constructs, stem cell culture, in vitro tissue modeling	ASCs	[89]
PEGDA, Irgacure 369	Photocurable, high-resolution structuring, RGD motif-presenting	2PP	Photocrosslinking	Bone tissue engineering, microstructured scaffolds	Ovine endothelial cells	[90]

## 3. Three-Dimensional Printing and Bioprinting Technologies for Cardiovascular Constructs: Strengths, Limitations, and Translational Perspectives

The selection of a suitable 3D printing or bioprinting technique critically influences the architectural fidelity, mechanical integrity, and biological performance of engineered cardiovascular constructs. Conventional 3D printing methods have substantially contributed to the fabrication of acellular scaffolds, vascular grafts, and patient-specific cardiovascular models, thereby supporting biomedical applications such as surgical planning, device testing, and implantable scaffold design. In parallel, 3D bioprinting has advanced as a transformative biofabrication approach that enables the spatially controlled deposition of living cells and biomaterials to create functional tissue constructs in vitro. These modalities span from extrusion-based printing to advanced microscale and support-based strategies, each offering distinct capabilities tailored to specific structural and functional requirements. Collectively, both conventional and bioprinting technologies provide complementary pathways toward translationally relevant cardiovascular constructs, bridging the gap between engineering design and clinical application [91,92]. Table 1 summarizes key advances in cardiovascular bioprinting and serves as a central reference for this field.

### 3.1. Extrusion-Based Bioprinting

**Figure 3:**
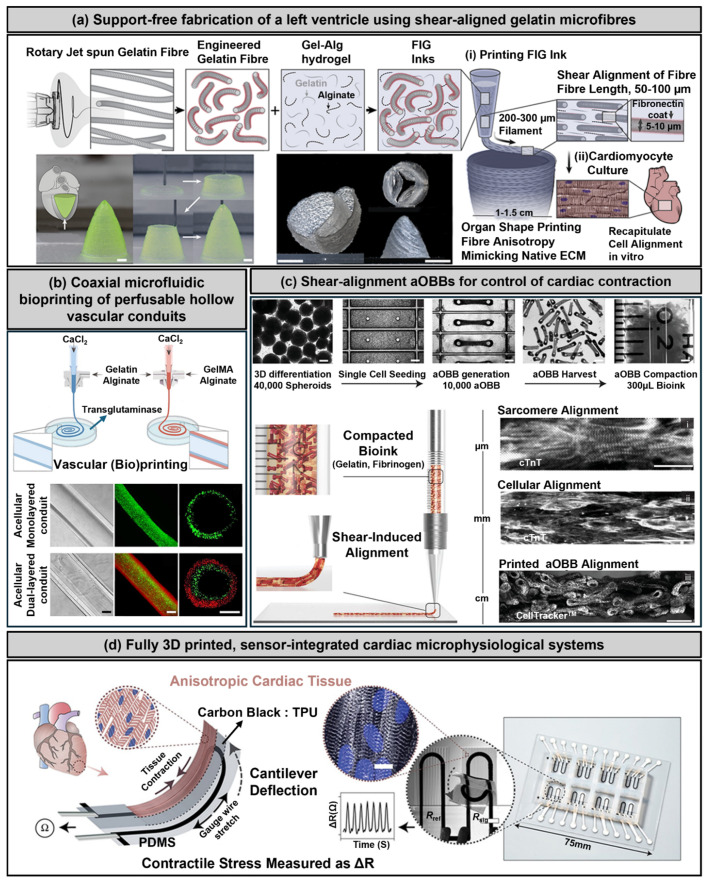
Extrusion-based 3D bioprinting (**a**) Fiber-infused gel scaffolds guide cardiomyocyte alignment in 3D-printed ventricles [scale bar = 2 mm (**left**), 5 mm (**right**)]. Adapted with permission from Ref. [7], Springer Nature. (**b**) Microfluidic bioprinting of tough hydrogel-based vascular conduits for functional blood vessels (scale bar = 200 μm). Adapted with permission from Ref. [94], The American Association for the Advancement of Science. (**c**) Programming cellular alignment in engineered cardiac tissue by bioprinting anisotropic organ building blocks [scale bar = 200 µm (**top-1**), 500 µm (**top-2,3,4**), 1000 µm. (**top-5**), 20 µm (**right-top**), 100 µm (**right-middle**), 2 mm (**right-bottom**)]. Adapted with permission from Ref. [95], John Wiley and Sons. (**d**) Instrumented cardiac microphysiological devices via multimaterial three-dimensional printing. Adapted with permission from Ref. [28], Springer Nature.

Extrusion-based bioprinting (EBB) remains the most widely employed technique in cardiovascular tissue engineering, primarily because of its compatibility with high-viscosity bioinks, capability of high-density cell encapsulation, and ease of scalability [36,68,93]. Utilizing pneumatic or mechanical force, the EBB continuously deposits cell-laden bioinks layer by layer through an extrusion nozzle, enabling the fabrication of anisotropic and volumetrically thick constructs (Figure 3).

Additionally, fiber-infused gel (FIG) bioinks, consisting of fibronectin-coated gelatin microfibers embedded within gelatin–alginate matrices, have been developed to replicate the native myocardium’s anisotropic extracellular matrix. Under shear stress during extrusion, the fibers align along the printing direction, significantly improving the shear-thinning behavior, elastic recovery, and cellular alignment. This approach successfully enabled support-free printing of anatomically relevant 3D cardiac ventricles exhibiting robust cardiomyocyte alignment, structural integrity, and electromechanical coupling (Figure 3a) [7].

EBB is especially advantageous in fabricating cardiac patches and vascular constructs with aligned cellular architecture using bioinks such as gelatin methacryloyl (GelMA) and decellularized extracellular matrix (dECM). Additionally, coaxial extrusion techniques employing core–shell configurations (e.g., alginate–GelMA blends) have successfully produced perfusable vascular channels, supporting endothelial cell lining and subsequent microvascular formation (Figure 3b) [94]. Despite their versatility, EBBs face inherent limitations, including a relatively low printing resolution and potential shear-induced cellular damage due to high extrusion pressures and shear forces. Recent efforts have addressed these challenges through the development of shear-thinning bioinks, the optimization of nozzle geometries, and tailoring printing parameters to enhance cell viability and structural precision [50].

Moreover, shear-alignment strategies that leverage anisotropic organ building blocks (aOBBs) and pre-aligned elongated microtissues composed of human iPSC-derived cardiomyocytes have been implemented. During extrusion, these aOBBs align further along the printing direction under shear and extensional forces, resulting in cardiac macrofilaments exhibiting multiscale structural coherence, from sarcomere-level alignment to tissue-scale organization. This approach significantly enhances contractile force generation and conduction velocities compared with conventional spheroid-based constructs (Figure 3c) [95].

Several advanced extrusion-based approaches have recently emerged to further improve construct functionality and fidelity. For instance, a multimaterial direct ink writing (DIW) platform has integrated soft PDMS substrates with embedded piezoresistive strain sensors. This system enables long-term, real-time monitoring of contractile stress and electrical anisotropy in engineered cardiac tissues derived from neonatal rat ventricular myocytes and human-induced pluripotent stem cell-derived cardiomyocytes (iPSC-CMs). The optimization of the filament spacing (~60 µm) facilitated precise sarcomere alignment and directional action potential propagation, closely replicating native myocardial properties (Figure 3d) [28].

Innovations also include robot-assisted in situ extrusion bioprinting, which enables the direct deposition of hydrogel-based bioinks onto patient-specific anatomical defects. Through non-planar path planning and automated fiducial marker registration, this approach has demonstrated sub-millimeter accuracy in reconstructing cranial bone defects. Such capabilities suggest substantial potential for translating this technique into cardiovascular surgery, facilitating real-time anatomically conformal tissue repair without the need for prefabricated implants [96].

Compared with other bioprinting modalities, EBB uniquely offers the capability to utilize highly viscous, cell-rich bioinks and continuously deposit thick, functionally relevant constructs. In contrast, inkjet bioprinting provides higher spatial resolution but is constrained by low cellular densities and frequent nozzle clogging. Photopolymerization-based techniques, such as stereolithography (SLA) and digital light processing (DLP), offer superior resolution and precision, but require specific photocurable bioinks, face limitations in bioink penetration depth, and introduce potential phototoxic effects. Conversely, EBB supports a wide array of bioinks, including multi-component and shear-thinning formulations, and seamlessly integrates strategies such as coaxial extrusion, fiber-based cellular alignment, and robot-assisted in situ bioprinting. These combined advantages establish EBB as the most versatile and widely adopted technique for engineering structurally and functionally sophisticated cardiovascular tissues, despite ongoing efforts to improve the printing resolution and reduce shear-induced cellular damage. Importantly, extrusion bioprinting has already advanced to early preclinical and even pilot clinical applications, such as vascular graft prototypes and bioprinted heart valves, highlighting its translational readiness compared with more experimental modalities. Nevertheless, achieving reproducibility under GMP conditions and minimizing shear-induced cell damage remain essential for regulatory approval.

### 3.2. Freeform Reversible Embedding of Suspended Hydrogels (FRESH)

Freeform reversible embedding of suspended hydrogels (FRESH) bioprinting is an advanced, support bath-based extrusion technique that enables the fabrication of soft, delicate hydrogels, such as collagen and decellularized extracellular matrix (dECM) [69,70,71,72,73]. FRESH utilizes a supportive gelatin-based slurry that transiently immobilizes printed bioinks during fabrication, preventing structural collapse and maintaining architectural fidelity [12,73]. After printing, the gelatin-based support bath was gently melted under mild physiological conditions, thereby preserving cell viability and maintaining the printed structural integrity [19]. This approach has demonstrated significant success in the high-resolution fabrication of anatomically precise cardiovascular tissues, including neonatal-scale cardiac ventricles and structurally accurate heart valves [9,19,74,75].

**Figure 4:**
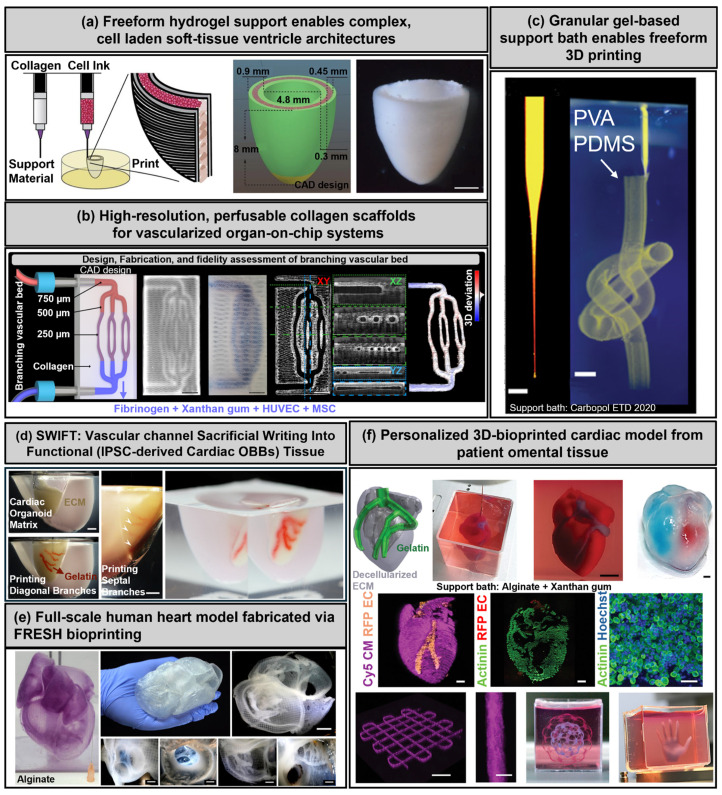
FRESH-based 3D bioprinting. (**a**) 3D bioprinting of collagen to rebuild components of the human heart (scale bar = 2 mm). Adapted with permission from Ref. [9], The American Association for the Advancement of Science. (**b**) 3D bioprinting of collagen-based high-resolution internally perfusable scaffolds for engineering fully biologic tissue systems (scale bar = 2 mm). Adapted with permission from Ref. [97], The American Association for the Advancement of Science. (**c**) Writing in the granular gel medium [scale bar = 1 mm (**left**), 3 mm (**right**)]. Adapted with permission from Ref. [100], The American Association for the Advancement of Science. (**d**) Biomanufacturing of organ-specific tissues with high cellular density and embedded vascular channels (scale bar = 5 mm). Adapted with permission from Ref. [12], The American Association for the Advancement of Science. (**e**) FRESH 3D bioprinting a full-scale model of the human heart (scale bar = 1 cm). Adapted with permission from Ref. [74], The American Chemical Society. (**f**) 3D printing of personalized thick and perfusable cardiac patches and hearts [scale bar = 0.5 cm (**top-left**), 1 mm (**top-right**), 1 mm (**bottom-left**), 100 µm (**bottom-right**)]. Adapted with permission from Ref. [29], John Wiley and Sons.

A recent advancement, known as FRESH v2.0, further enhanced this technique by enabling the direct bioprinting of unmodified collagen at an unprecedented resolution (approximately 20 µm). This refinement allowed for the precise fabrication of neonatal-scale ventricles and patient-specific heart valves, capturing fine anatomical details and significantly advancing efforts toward creating clinically viable cardiac constructs (Figure 4a) [9].

Building upon the foundational capabilities of FRESH, an advanced bioprinting strategy was devised to facilitate the fabrication of collagen-based, high-resolution, internally perfusable scaffolds (CHIPS), thereby significantly enhancing the architectural complexity and functional fidelity of engineered tissue systems. By refining material tolerancing and utilizing custom multi-material FRESH bioprinters, the platform achieved microscale control over both positive and negative spaces within perfusable networks, achieving feature resolution of less than 20 μm. These collagen-I-based constructs were printed within a thermo-reversible gelatin support bath, which enabled precise deposition and nondestructive retrieval. Furthermore, a specialized perfusion bioreactor system was implemented to sustain dynamic culture and maintain structural integrity during flow. Quantitative optical coherence tomography and 3D gauging analyses demonstrated that FRESH-printed CHIPS consistently reproduced complex microvascular geometries with minimal deviation (<11 μm), including circular lumens as small as 250 μm in diameter. The system also supported molecular weight-dependent diffusion, spatial ECM patterning, and multi-cellular vascular network formation, illustrating the versatility of FRESH in generating fully biologic, perfusable tissue architectures suitable for translational applications (Figure 4b) [97].

In addition to these advancements, a recently reported strategy, termed tunable rapid assembly of collagenous elements (TRACE), introduced a macromolecular crowding-based approach to accelerate the liquid–gel transition of unmodified collagen, thereby enabling its direct use as a high-fidelity bioink for bioprinting [98]. This method facilitated the rapid fabrication of collagen constructs across multiple length scales, from microscale features to macroscale tissues, while preserving physiological biocompatibility and supporting cell self-assembly and morphogenesis. By allowing pH-neutral and structurally versatile collagen printing with unprecedented throughput, TRACE substantially broadens the scope of FRESH bioprinting, providing a robust pathway to generate anatomically complex and biologically functional cardiovascular tissue models.

In addition to microscale and mesoscale demonstrations, a landmark study extended the capabilities of FRESH to fabricate an entire full-scale human heart model, reconstructed directly from high-resolution MRI data [99]. By translating voxelated imaging datasets into printing instructions, the approach enabled the layer-by-layer deposition of collagen-based bioinks within a gelatin support bath, achieving sub-millimeter fidelity across anatomically intricate features such as chordae tendineae, septa, and valve leaflets. The printed constructs exhibited native-like biomechanical properties and retained compatibility with histological validation techniques, underscoring both the scalability and translational relevance of FRESH for organ-level cardiovascular bioprinting. This achievement highlights the potential of support bath-based bioprinting not only for functional tissue constructs but also for anatomically precise organ-scale models, bridging the gap between experimental demonstrations and future clinical applications.

One of the earliest foundational demonstrations of support bath-based bioprinting involved the deposition of materials into soft granular gel media, facilitating the stable fabrication of intricate three-dimensional structures with minimal constraints from gravity, surface tension, or material viscosity. In this method, a microscale injection tip locally fluidizes a jammed hydrogel particle matrix—typically composed of Carbopol microgels—during the material deposition process. As the tip advances, the gel rapidly resolidifies in its wake, effectively trapping the injected material with remarkable spatial precision. This physical confinement mechanism enabled the printing of diverse materials, including hydrogels, polymers, colloids, and even living cells, into high-aspect-ratio geometries and thin-walled hollow shells without the need for additional support structures. Notably, this technique facilitated the creation of stable, freestanding architectures, such as helically patterned rods, vascular-like tubular networks, and encapsulated multilayered constructs. Structures fabricated from photocrosslinkable polymers were successfully retrieved from the support bath post-printing, while uncrosslinked colloidal systems remained stably suspended for extended periods. The use of a physically reversible granular medium provided high fidelity and long working times, laying the groundwork for subsequent refinements of support bath bioprinting strategies such as FRESH (Figure 4c) [100].

Moreover, hybrid bioprinting strategies that integrate FRESH with coaxial extrusion methods have been developed to construct perfusable vascular channels embedded within dense cardiac tissues. This integration effectively improved tissue viability and electromechanical coupling by ensuring adequate nutrient transport and promoting robust vascularization within volumetrically thick constructs. Such combined approaches illustrate the versatile applicability of FRESH in producing structurally complex, multi-cellular cardiac tissues with embedded functional vasculature (Figure 4d) [12]. Despite these substantial advantages, the FRESH technique still faces notable challenges. Key limitations include the complexity of post-processing procedures, potential cytotoxic effects from residual support bath components, and stringent thermal control requirements critical for preserving cellular viability during support removal. Overcoming these challenges is essential for scaling up FRESH-based constructs toward clinical applications.

By expanding the scale and anatomical realism of bioprinted constructs, FRESH was further applied to fabricate a full-sized, structurally accurate model of the human heart, representing a critical milestone in organ-scale bioprinting. By leveraging high-resolution magnetic resonance imaging (MRI) data and translating it into voxelated printing instructions, the entire organ—comprising intricately aligned muscle walls, septa, valves, and outflow tracts—was reconstructed layer by layer using collagen bioinks within a gelatin support bath. The method achieved sub-millimeter fidelity across the heart’s geometry, accurately replicating fine structural features, such as chordae tendineae and valve leaflets. Importantly, the printed constructs retained their native-like biomechanical properties and were compatible with standard histological staining techniques, enabling detailed structural validation. This study underscored the scalability of FRESH, not only for fabricating microscale vascular features, but also for reproducing macro-anatomical structures essential for surgical planning, educational modeling, and future regenerative medicine applications (Figure 4e) [74].

Utilizing native ECM-based bioinks, FRESH facilitates the printing of biologically complex materials with unmatched anatomical precision. For example, vascularized cardiac patches and miniaturized heart constructs printed using autologous cardiomyocytes and dECM bioinks have exhibited spontaneous cellular alignment, robust vascular network formation, and physiologically relevant calcium transients, highlighting the potential for personalized cardiac regeneration (Figure 4f) [29].

Compared to conventional extrusion-based bioprinting (EBB), which excels in handling viscous bioinks and providing structural robustness, FRESH uniquely enables the printing of low-viscosity, ultra-soft hydrogels and native ECM-based bioinks with exceptional precision. Additionally, unlike digital light processing (DLP) bioprinting, which is restricted to photopolymerizable bioinks and predominantly planar geometries, FRESH supports complex, freeform, and patient-specific three-dimensional constructs, incorporating integrated perfusable vascular networks. Therefore, FRESH effectively addresses critical gaps in bioprinting technology, specifically in producing delicate, biologically active, and anatomically intricate cardiovascular tissues typically challenging to fabricate with traditional bioprinting methods. FRESH uniquely enables fabrication of full-scale, anatomically precise heart models using collagen, positioning it as a promising tool for patient-specific surgical planning and education. However, translation toward implantable cardiovascular tissues requires overcoming the challenges of support material removal, process standardization, and compliance with ISO 10993 hemocompatibility and thrombogenicity evaluations.

### 3.3. Stereolithography and Digital Light Processing

Stereolithography (SLA) and digital light processing (DLP) are advanced photopolymerization-based bioprinting techniques that utilize light sources to crosslink photocurable bioinks, achieving highly precise and structurally complex architectures. These modalities enable the fabrication of finely patterned scaffolds at microscale resolution, effectively replicating the intricate microstructural features of native cardiovascular tissues [16,83,84,85,86,87]. Photocurable biomaterials such as gelatin methacryloyl (GelMA) [16,83,84,85,86,87] and polyethylene glycol diacrylate (PEGDA) [51,88] have been extensively employed in these methods due to their tunable mechanical properties, rapid polymerization kinetics, and biocompatibility [44].

**Figure 5:**
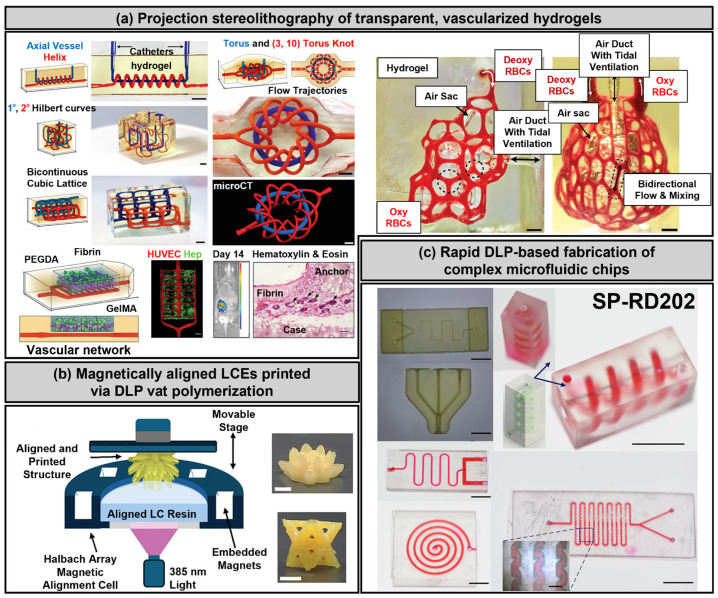
SLA-based 3D bioprinting. (**a**) Multivascular networks and functional intravascular topologies within biocompatible hydrogels [scale bar = 3 mm (**top-left**), 1 mm (**top-right**), 1 mm (**bottom**)]. Adapted with permission from Ref. [103], The American Association for the Advancement of Science. (**b**) Digital light process 3D printing of magnetically aligned liquid crystalline elastomer free-forms [scale bar = 3 mm (**top**), 5 mm (**bottom**)]. Adapted with permission from Ref. [104], John Wiley and Sons. (**c**) 3D printing of individualized microfluidic chips with DLP-based printer [scale bar = 5 mm, 400 µm (channel)]. Adapted with permission from Ref. [105], MDPI.

DLP, in particular, utilizes spatially patterned light exposure through digital micromirror device (DMD) systems to achieve rapid, layer-by-layer crosslinking with minimal shear stress on encapsulated cells. This capability makes DLP especially suited for fabricating geometrically sophisticated cardiac microtissues, including perfusable microvascular networks and highly organized myocardial architectures. However, critical challenges such as phototoxicity, limited penetration depth of light, and potential sedimentation-induced uneven cell distribution within printed layers must be carefully managed to maintain cellular viability and construct homogeneity [101,102]. Recent advances have further refined DLP methodologies by employing visible-light DMD systems, which significantly enhance biocompatibility by reducing the cytotoxic effects typically associated with UV-based photopolymerization. For instance, high-resolution, perfusable GelMA constructs with spatially defined populations of cardiomyocytes and stromal cells have demonstrated improved cellular viability, robust functional coupling, and precise architectural control. Such innovations underscore the strong potential of DLP-based bioprinting for creating sophisticated, functionally relevant cardiac constructs (Figure 5a) [103]. In addition, emerging approaches utilizing physical field-assisted patterning during photoprinting have been developed to enhance structural alignment and anisotropic organization. An example is the magnetic field-assisted digital light processing (DLP) strategy, which utilizes liquid crystalline elastomers (LCEs) to enable spatially controlled alignment within intricate, freeform three-dimensional structures through a regulated magnetic field (e.g., 100 mT Halbach array). Although initially demonstrated in soft robotic applications, this magnetically guided alignment technique holds significant potential for cardiac tissue engineering, where anisotropic architecture and synchronized contractility are essential for functional integration and performance (Figure 5b) [104]. Additionally, recent studies have underscored the utility of DLP in the rapid fabrication of precise microfluidic systems. It has been demonstrated that DLP can directly print enclosed microchannels with dimensions exceeding 200 µm with remarkable accuracy. To address challenges related to resin blockage in finer geometries, an innovative open-channel design was proposed, subsequently sealed with transparent tape. Despite the inherent moderate cytotoxicity associated with unmodified resins, GelMA surface coatings significantly enhanced biocompatibility. This method successfully produced shear stress microchips and spiral microchannel devices, confirming DLP’s versatility and speed for biomedical prototyping and cardiovascular applications (Figure 5c) [105]. These advancements position SLA and DLP as compelling alternatives to conventional extrusion-based bioprinting (EBB). While EBB excels in volumetric tissue deposition and high-density cell encapsulation, it remains limited by its lower spatial resolution and susceptibility to shear-induced cellular damage. In contrast, DLP uniquely facilitates high-resolution, cell-friendly fabrication of microscale patterned architectures, enabling precise control over multiple cell types, mechanical gradients, and tissue complexity within a single construct. The increasing availability of visible light-compatible bioinks, alongside ongoing innovations addressing penetration depth, photoinitiator biocompatibility, and dynamic cell alignment, further reinforces the promise of SLA and DLP for engineering anatomically accurate and functionally sophisticated cardiovascular tissues. While SLA and DLP demonstrate excellent resolution and perfusable microvascular architectures, they remain largely confined to preclinical proof-of-concept studies. Future translation will depend on the development of visible light-compatible, FDA/EMA-approvable bioresins and reproducible large-scale manufacturing protocols.

### 3.4. Melt Electrowriting

Although melt electrowriting (MEW) is conventionally categorized as a high-resolution 3D printing technique, in cardiovascular tissue engineering, it has been increasingly employed in hybrid strategies where MEW-derived scaffolds are combined with bioinks and cell-laden hydrogels. In this review, MEW is therefore discussed specifically in the context of its integration with bioprinting approaches to enhance structural anisotropy, cell alignment, and vascularization. Melt electrowriting (MEW) uniquely combines principles of electrospinning and fused deposition modeling, enabling the precise deposition of fine, micron-scale fibers from molten thermoplastic polymers under the influence of a high-voltage electric field. MEW has gained substantial attention in cardiovascular tissue engineering due to its ability to fabricate highly controlled, anisotropic scaffolds that effectively mimic the native fibrous architecture of myocardial and vascular tissues [59,106].

MEW-derived scaffolds have shown particular promise in cardiovascular applications by promoting directional cardiomyocyte alignment, enhanced sarcomeric organization, and the formation of continuous endothelial monolayers within engineered microchannels. These characteristics are crucial for developing multilayered, mechanically robust cardiac patches capable of sustaining physiologically relevant contractile forces. However, current limitations of MEW include a restricted range of suitable thermoplastic materials (primarily polycaprolactone (PCL)) [76,77,78,79,80,81,82], the requirement for meticulous thermal control during printing, and limited bioactivity of the printed scaffolds, necessitating additional surface modifications or coatings to improve cellular interactions. Recent innovations have expanded the applicability of MEW through advanced printing strategies. An example is the development of a print-and-fuse approach, in which sacrificial microfibers printed via MEW are subsequently embedded within elastomeric matrices to generate perfusable biomimetic microfluidic networks. Although primarily applied to elastomer-based microfluidics rather than direct cardiac cell encapsulation, this strategy underscores MEW’s precision in guiding complex vascular architectures and potential applications for fabricating vascularized cardiac tissues with hierarchical, controlled perfusion pathways [107].

**Figure 6:**
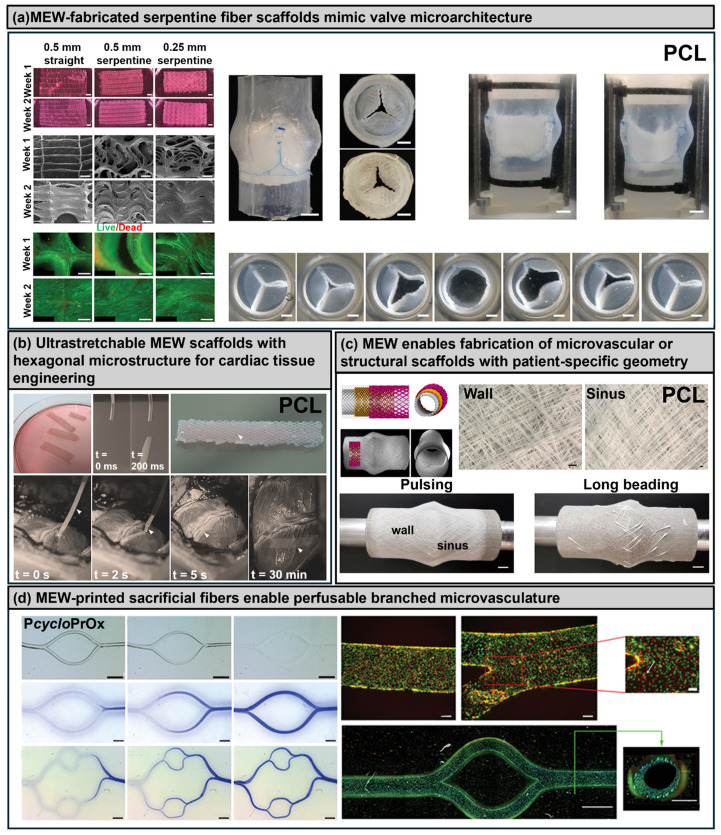
Melt electrowriting-based 3D bioprinting. (**a**) Biologically inspired scaffolds for heart valve tissue engineering via melt electrowriting [scale bar = 2 mm (**left-top**), 500 µm (**left-bottom**), 200 µm (**left-bottom**), 5 mm (**right**)]. Adapted with permission from Ref. [76], John Wiley and Sons. (**b**) Melt electrowriting allows for the tailored microstructural and mechanical design of scaffolds to advance functional human myocardial tissue formation. Adapted with permission from Ref. [108], John Wiley and Sons. (**c**) Melt electrowriting of complex 3D anatomically relevant scaffolds [scale bar = 200 μm (**top**), 5 mm (**bottom**)]. Adapted with permission from Ref. [109], Elsevier. (**d**) Print-and-fuse strategy for sacrificial filaments enables biomimetically structured perfusable microvascular networks with functional endothelium within 3D hydrogels [scale bar = 1 mm (**left**), 100 μm (**right-top**), 1 mm (**right-bottom**)]. Adapted with permission from Ref. [107], John Wiley and Sons.

Furthermore, MEW has successfully produced anatomically and mechanically biomimetic scaffolds tailored for heart valve tissue engineering. Through controlled fiber orientation and precise layering techniques, scaffolds replicating the trilayered fibrosa–spongiosa–ventricularis structure of native aortic valves were created. These constructs demonstrated distinct, region-specific mechanical properties, supported robust valve interstitial cell infiltration, and promoted extracellular matrix deposition, highlighting MEW’s capacity to recapitulate complex, multilayered structural anisotropy essential for dynamic cardiovascular tissues such as heart valves [76] (Figure 6a). Complementary techniques, such as focused rotary jet spinning (FRJS), have also demonstrated potential in fabricating functional cardiac structures by rapidly generating anisotropic fiber scaffolds without the need for direct-write methods. FRJS-produced “FibraValves,” for instance, exhibited biomimetic extracellular matrix architectures, suitable mechanical properties, and effective cellular infiltration in vivo, emphasizing that fiber-based scaffold fabrication methods like MEW and FRJS hold significant potential for clinically relevant cardiac tissue engineering [48].

To further capitalize on the architectural tunability of MEW, research has demonstrated the integration of controlled fiber microarchitectures with tissue-specific mechanical properties tailored for cardiovascular applications. Using design-driven printing strategies, microfibrous scaffolds were engineered with spatially defined variations in fiber alignment, density, and orientation to emulate the structural anisotropy of native cardiac tissues. This approach facilitates the fabrication of constructs exhibiting distinct mechanical gradients and direction-dependent stiffness, which are critical for replicating the biomechanical environment of the heart and blood vessels. Additionally, combining MEW with elastomeric or hydrogel substrates enables the generation of composite scaffolds that merge microscale topographical cues with bulk mechanical compliance. Such constructs not only preserve high-resolution fiber alignment but also support effective cell adhesion, proliferation, and extracellular matrix remodeling. These findings underscore the capacity of MEW to transcend uniform scaffold fabrication, allowing for the design of biomimetic, spatially heterogeneous tissue constructs that more accurately reflect the complexity of cardiovascular tissues (Figure 6b) [108]. In addition to mechanical mimicry, MEW has also been utilized to create biofunctional scaffolds that enhance cellular organization and vascularization within engineered cardiac tissues. By fabricating aligned microfibrous scaffolds with embedded microgrooves or patterned topographies, researchers have successfully directed cardiomyocyte elongation, sarcomere formation, and synchronous contractions. These structural cues have been shown to significantly enhance electrical signal propagation and mechanical coupling across the tissue construct. Moreover, MEW has been effectively integrated with hydrogel casting techniques to develop dual-phase constructs, where the fibrous scaffold provides mechanical reinforcement, whereas the hydrogel phase supports cellular encapsulation and diffusion. This hybrid strategy enabled the formation of prevascularized myocardial patches containing both endothelial and cardiac cell types, which exhibited early lumen formation and angiogenic potential. These results illustrate how MEW can be leveraged not only for precision microfabrication but also for guiding cell–matrix interactions critical to cardiovascular regeneration, particularly when combined with cell-laden hydrogels or biomimetic extracellular matrix environments (Figure 6c) [109]. A recent advancement in MEW-based scaffold design introduced a print-and-fuse strategy, wherein sacrificial thermoplastic microfibers fabricated by MEW are embedded within elastomeric matrices and subsequently dissolved to generate complex, perfusable microchannel networks. This method allowed for the construction of vascular architectures with hierarchical organization and spatial fidelity, closely mimicking the branching patterns and lumen dimensions of the native microvasculature. By tuning the filament geometry and embedding process, the resulting microfluidic constructs exhibited biomimetic flow dynamics and structural robustness. Importantly, the fused constructs supported endothelialization and maintained long-term patency under perfusion, demonstrating their potential as platforms for vascularized tissue. Although this approach was primarily applied using elastomeric materials, its successful integration with MEW underscores the capacity of the method to guide perfusable microchannel formation with microscale precision, offering new opportunities for engineering vascularized cardiac tissues and organ-on-chip systems (Figure 6d) [107].

Compared to extrusion-based bioprinting (EBB), which excels in bulk hydrogel deposition and high-density cell encapsulation but lacks microscale fiber precision, MEW provides unparalleled control over fiber placement and structural anisotropy. Unlike photopolymerization-based methods such as digital light processing (DLP) or stereolithography (SLA), MEW does not require photoinitiators and produces mechanically robust thermoplastic-based scaffolds that provide enduring structural support. While photopolymerization excels in creating fine-resolution, hydrogel-based vascular networks, MEW’s capability to fabricate precise microfibrous scaffolds allows for integration with soft hydrogel matrices, facilitating hierarchical vascularization, structural reinforcement, and enhanced nutrient diffusion. Therefore, MEW serves as a critical complementary approach to existing bioprinting modalities, offering precise and mechanically robust fiber frameworks essential for long-term stability, cellular alignment, and hierarchical vascular integration in advanced cardiovascular tissue engineering strategies. MEW’s capacity to fabricate mechanically robust scaffolds closely mimicking fibrous extracellular matrices makes it highly attractive for cardiovascular grafts and heart valves, areas where structural integrity is paramount. Its use of FDA-cleared polymers such as PCL further enhances its translational potential, although bioactivity must be addressed via surface functionalization.

### 3.5. Two-Photon Polymerization

Two-photon polymerization (TPP) is an advanced photopolymerization technique that uses femtosecond pulsed lasers to initiate polymerization with submicron resolution through nonlinear two-photon absorption processes [89,90]. This ultrahigh-resolution capability renders TPP particularly suitable for engineering microscale cardiac and vascular architectures with precisely defined spatial properties, including microvascular geometries and stiffness gradients [107,110]. Owing to its inherent spatial selectivity, where polymerization occurs exclusively at the laser’s focal volume, TPP uniquely enables the fabrication of arbitrary three-dimensional structures without the need for masks or supporting structures. TPP has demonstrated significant potential in cardiovascular tissue engineering, especially within microfluidic heart-on-a-chip platforms and complex vascularized microenvironments.

**Figure 7:**
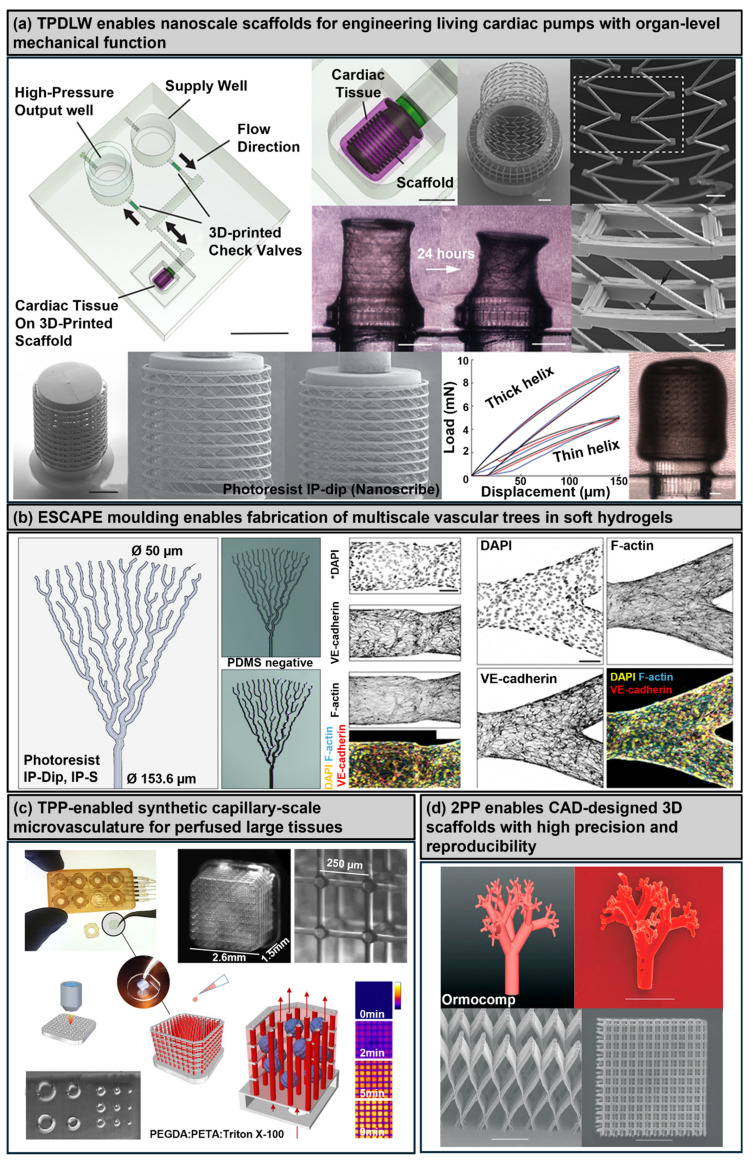
2PP-based 3D bioprinting. (**a**) Engineering a living cardiac pump on a chip using high-precision fabrication (scale bar = 5 mm (**top-left**), 1 mm (**top-middle-left**), 200 µm (**top-middle-right**), 500 µm (**middle-middle**), 50 µm (**right-top-middle**), 500 µm (**bottom-left**), 200 µm (**bottom-middle-right**). Adapted with permission from Ref. [32], The American Association for the Advancement of Science. (**b**) Sacrificial capillary pumps to engineer multiscalar biological forms (scale bar = 100 µm). Adapted with permission from Ref. [111], Springer Nature. (**c**) Large-scale perfused tissues via synthetic 3D soft microfluidics. Adapted with permission from Ref. [112], Springer Nature. (**d**) Two-photon polymerization technique for microfabrication of CAD-designed 3D scaffolds from commercially available photosensitive materials [scale bar = 40 µm (**top-right**), 90 µm (**bottom**), 7 µm (**bottom**)]. Adapted with permission from Ref. [113], John Wiley and Sons.

Two-photon direct laser writing (TPDLW), a variant of two-photon polymerization (TPP), has recently been utilized to fabricate miniaturized functional cardiac pump-on-a-chip platforms that accurately replicate the biomechanics of ventricular tissues. By employing nanoscale precision, scaffolds with adjustable stiffness, auxetic (negative Poisson’s ratio), or helical geometries were produced to facilitate the cyclic contraction and robust formation of human induced pluripotent stem cell (hiPSC)-derived ventricular chambers. Furthermore, microscale suspension valves fabricated via TPDLW provided unidirectional flow rectification under physiological pressures, enabling the integration of microfluidic circuits capable of generating physiologically relevant pressure–volume loops. Such innovations underscore the remarkable potential of TPP and TPDLW in recreating the biomechanical complexity, structural fidelity, and functional integration required for advanced cardiovascular organ-on-chip systems (Figure 7a) [32].

Recent advancements in two-photon polymerization have facilitated the creation of hierarchically perfusable microvascular architectures embedded within soft hydrogel matrices that closely replicate the physiological organization of the native vascular networks. By utilizing custom-formulated photopolymerizable hydrogels and the voxel-by-voxel precision of two-photon laser scanning, researchers have constructed vascular networks with multiscale branching, lumen diameters of less than 50 μm, and open perfusion connectivity within biocompatible matrices. These vascular topologies were seamlessly integrated with the surrounding tissue compartments and demonstrated the ability to support sustained media flow and long-term cellular viability even in metabolically demanding tissues. Notably, this approach enables the functional vascularization of centimeter-scale constructs, thereby overcoming the limitations associated with diffusion-mediated nutrient transport in thick engineered tissues. By programming the microvascular topology to replicate hierarchical tree-like and looped flow geometries, the fabricated networks provide enhanced flow distribution and oxygen delivery, highlighting the potential of two-photon polymerization for engineering physiologically relevant vascularized tissue systems. These innovations represent a significant advancement toward organ-scale tissue fabrication with integrated vasculature capable of functional perfusion and metabolic support (Figure 7b) [111].

In cardiovascular tissue applications, TPP has been employed to construct high-resolution three-dimensional microvascular networks using custom, non-swelling PEG-based photoresins. This strategy enabled direct printing of capillary-sized channels (10–70 µm in diameter) within soft microfluidic grids, which were subsequently integrated into perfusion chips to sustain multi-millimeter-scale tissue constructs. Such precisely fabricated microvascular networks successfully supported long-term perfusion, effectively prevented hypoxic conditions and apoptosis, and significantly enhanced differentiation outcomes in neural and hepatic tissue cultures compared with traditional organoid models lacking perfusion (Figure 7c) [112].

Recent studies utilizing TPP have successfully fabricated instructive microstructures, such as hemisphere-shaped microcages, trabecular bone-mimicking lattices, and anisotropic cardiac scaffolds, providing controlled topographical cues that effectively guide cell morphology, differentiation pathways, and tissue-specific alignment. Biomaterials commonly employed in TPP-based approaches include gelatin, bovine serum albumin (BSA), and hybrid synthetic resins, such as Ormocer^®^ or polyethylene glycol diacrylate (PEGDA), owing to their tunable stiffness, biocompatibility, and ability to support detailed microstructural fidelity. Despite its unparalleled precision, the TPP currently faces challenges, such as relatively low throughput, limited scalability, and significant system complexity, which restrict its use primarily to small-scale constructs or complementary roles within multimodal bioprinting platforms. Current advances, including the use of spatial light modulators and parallel beam splitting, are being actively explored to address these limitations and significantly enhance the fabrication throughput via simultaneous multispot polymerization (Figure 7d) [113].

Each bioprinting modality offers distinctive advantages: extrusion-based bioprinting (EBB) enables the scalable fabrication of cell-dense volumetric constructs, stereolithography/digital light processing (SLA/DLP) facilitates rapid and precise microscale patterning, melt electrowriting (MEW) excels in anisotropic fiber deposition, FRESH facilitates the printing of ultrasoft biologically complex tissues, and TPP uniquely delivers unparalleled submicron topographical precision. The strategic integration of TPP-fabricated microscale scaffolds with complementary bioprinting modalities, dynamic stimulation systems, and real-time sensing technologies is anticipated to significantly advance cardiovascular tissue engineering, ultimately enabling the comprehensive replication of the complex structural hierarchy and functional dynamics characteristics of native cardiac and vascular tissues [114].

### 3.6. Challenges and Mitigation Strategies

Despite the rapid progress of bioprinting modalities, several recurring pitfalls must be considered to ensure reproducibility and safety. For example, in digital light processing (DLP), the use of UV-based photoinitiators can induce cytotoxic effects, which can be mitigated by employing visible light photoinitiators or improved resin formulations. Melt electrowriting (MEW) may cause thermal degradation of polymers, which can be mitigated through material pre-selection (e.g., PCL), mild processing conditions, or post-functionalization strategies. Extrusion bioprinting often exposes cells to high shear stress, but this can be minimized by using shear-thinning bioinks, optimizing nozzle geometry, and reducing extrusion pressures. These representative strengths and limitations of each modality are summarized in Table 2, which provides a comparative overview. Collectively, these approaches enhance constructive fidelity and preserve cellular viability, facilitating more reliable and translationally relevant outcomes. Beyond technical challenges, a critical bottleneck remains the translation of these modalities under GMP-compliant conditions. Sterilization, reproducibility, and large-scale manufacturing standards must be met to transition from research-grade constructs to clinically approved therapies. Moreover, cardiovascular applications specifically require ISO 10993-compliant hemocompatibility and thrombogenicity testing, which will serve as essential regulatory checkpoints for future clinical trials.

## 4. Challenges in Cardiovascular Bioprinting

Despite remarkable advances in 3D bioprinting technologies, critical challenges still impede the successful fabrication of fully functional and clinically translatable cardiovascular tissues. These limitations primarily involve achieving functional vascularization; ensuring sufficient mechanical stability; maintaining long-term tissue functionality; and facilitating effective host integration, each presenting distinct biological, technological, and translational barriers.

### 4.1. Achieving Functional Vascularization

One of the foremost challenges in engineering thick and viable cardiovascular tissues is the establishment of functional, perfusable vasculature. Owing to inherent diffusion limitations, oxygen and nutrient supply typically cannot extend beyond approximately 100–200 μm from the nearest vascular channel, making vascularization crucial for maintaining tissue viability in thicker constructs [14].

Current strategies, including sacrificial templating using gelatin-based bioinks [19], coaxial extrusion-based channel fabrication [18], and microfluidic fiber printing methods [20], offer promising partial solutions. However, these approaches still face significant constraints related to limited spatial resolution, inadequate perfusion efficacy, scalability limitations, and challenges in achieving hierarchical vascular architecture. Moreover, several existing vascularization methods depend on post-printing endothelial cell seeding or spontaneous endothelial cell self-assembly. Such approaches often fail to replicate the structural hierarchy, lumen patency, and endothelial barrier integrity necessary for physiological vascular function under sustained shear stress conditions. Although natural bioinks such as fibrin, decellularized extracellular matrix (dECM), and gelatin methacryloyl (GelMA) actively support angiogenic sprouting, the long-term maintenance of functional lumens and robust endothelial barrier function remains challenging [35,37]. Although microfluidic integration into bioprinted constructs has improved vessel geometry control and shear stress management [25], further refinement is necessary to reliably achieve perfusion at clinically relevant organ-scale dimensions [28]. Recent breakthroughs in two-photon polymerization-based (TPP)-based fabrication have successfully demonstrated perfusable capillary-scale microvascular networks, providing a promising pathway toward achieving precise and dense microvascular patterning within densely constructed cells [115]. Nonetheless, translating these highly precise but small-scale strategies into larger clinically relevant engineered tissues remains a significant challenge.

### 4.2. Ensuring Mechanical Stability

Engineered cardiovascular constructs are subjected to constant dynamic mechanical forces, such as cyclic myocardial contractions and pulsatile vascular blood flow. Therefore, printed tissues must exhibit appropriate elasticity, viscoelastic behavior, fatigue resistance, and structural integrity to withstand sustained biomechanical loading. Despite their excellent bioactivity, natural biomaterials such as gelatin and collagen often degrade prematurely under mechanical stress, significantly limiting their suitability for long-term functional applications [45]. Conversely, synthetic polymers (e.g., as PEGDA and polycaprolactone (PCL)) offer superior mechanical durability but lack intrinsic bioactivity, cell adhesion, and tissue integration properties, thus necessitating bioactive functionalization or hybrid bioink formulations [52]. Advanced strategies employing double-network hydrogels and scaffolds reinforced via melt electrowriting (MEW) have shown notable improvements in stiffness, durability, and toughness, closely approximating the elastic properties of native myocardial tissue (10–20 kPa) [54,59]. Recent research leveraging dual-layered MEW-GelMA composites successfully promoted anisotropic cardiomyocyte alignment and significantly enhanced cardiac contractile force, underscoring the importance of strategic biomaterial combinations to achieve mechanical robustness, along with biological functionality [59]. Nevertheless, achieving fully synchronized electromechanical coupling and long-term mechanical resilience that precisely replicates the anisotropic and hierarchical characteristics of native cardiac tissues remains an unmet need [61,116].

### 4.3. Maintaining Long-Term Functionality

Sustaining phenotypic stability and achieving mature functional performance in bioprinted cardiovascular tissues over extended periods are major ongoing challenges. Printed cells often exhibit immature or dedifferentiated phenotypes owing to inadequate biomechanical, topographical, or biochemical stimuli provided by the engineered microenvironment [40,117].

In cardiac constructs, suboptimal cell alignment or insufficient electrical conductivity frequently leads to asynchronous contractions, arrhythmias, and diminished functional outputs. Although conductive bioinks, such as carbon nanotube (CNT)–GelMA composites and micropatterned scaffolds, have successfully enhanced electrical coupling and signal propagation, fully restoring adult-level excitation–contraction coupling and consistent synchronous contractions remains challenging [46,48]. Therefore, long-term strategies integrating continuous electrical stimulation, mechanical conditioning, and tailored biochemical cues are required to promote sustained cellular maturation and function.

### 4.4. Promoting Functional Engraftment

Even when engineered cardiovascular constructs demonstrate adequate mechanical stability and biological functionality, promoting effective engraftment with host tissue remains a critical challenge. Functional engraftment failures often result from immune-mediated rejection, mechanical mismatches between engineered constructs and native tissues, and insufficient vascular inosculation, ultimately compromising the long-term performance and viability.

To address these limitations, strategies leveraging autologous cell sources, immune-modulating or immune-evasive biomaterials, and precisely controlled cytokine-enriched microenvironments have been explored, showing considerable potential for enhancing biocompatibility, reducing inflammatory responses, and mitigating fibrotic encapsulation [118,119]. Nevertheless, successful engraftment also critically depends on seamless vascular and mechanical integration, ensuring the robust anastomosis of bioprinted microvascular networks with host vasculature, as well as mechanical compatibility, to support physiological loading conditions.

Despite these promising advancements, rigorous validation using large-animal models remains limited, and clear regulatory frameworks for translating sophisticated bioengineered constructs into clinical practice are yet to be fully established. Addressing these translational gaps through systematic preclinical evaluation and developing comprehensive regulatory pathways is essential for facilitating successful clinical translation and ensuring reliable functional engraftment of engineered cardiovascular tissues [120].

## 5. Applications of 3D-Bioprinted Cardiovascular Tissues

The advent of 3D bioprinting technologies has significantly advanced the fields of regenerative medicine, drug discovery, and disease modeling. Through the integration of precise biofabrication methods, stem cell technologies, and advanced biomaterials, 3D-bioprinted cardiovascular tissues have achieved greater biological relevance and potential clinical utility. The applications of bioprinted tissues can be broadly categorized into clinical therapeutic approaches and preclinical research models.

### 5.1. Clinical and Therapeutic Applications

Bioprinted cardiovascular constructs are being actively developed for therapeutic applications, including cardiac patches, vascular grafts, and injectable implants. For example, bioprinted cardiac patches have shown promise in myocardial infarction therapies by promoting angiogenesis, restoring contractile function, and reducing adverse fibrotic remodeling. Specifically, cardiac patches composed of gelatin methacryloyl (GelMA) and cardiac decellularized extracellular matrix (dECM) loaded with cardiac progenitor cells demonstrated enhanced cellular retention, functional engraftment, and integration within the host myocardium in vivo [40]. Similarly, hybrid elastic scaffolds combining polycaprolactone (PCL) and poly(glycerol sebacate) (PGS) were developed to match the mechanical characteristics of native cardiac tissues, thereby providing a supportive environment for cardiac regeneration and functional recovery [118]. Bioprinted vascular grafts engineered via coaxial extrusion or melt electrowriting (MEW) have facilitated the fabrication of perfusable, mechanically robust, and small-diameter vascular conduits. These constructs have demonstrated improved long-term patency rates, endothelialization efficiency, and mechanical resilience, thereby showing significant promise for clinical vascular replacement applications [46,121]. Additionally, bioprinted constructs are being increasingly explored as controlled drug delivery platforms. For example, nanocellulose-based cardiac patches incorporating curcumin-loaded polymeric carriers have been developed for localized, sustained anti-inflammatory treatment after myocardial infarction, highlighting their dual therapeutic and regenerative potential.

### 5.2. Toward Whole-Organ Bioprinting

Although complete bioprinting of functional human-scale cardiovascular organs remains a long-term goal, recent breakthroughs have demonstrated critical feasibility. Advanced techniques, such as freeform reversible embedding of suspended hydrogels (FRESH), have successfully enabled bioprinting of anatomically precise neonatal-scale collagen heart constructs, preserving critical internal trabecular structures and microarchitectural features [9]. Additionally, recent studies have demonstrated small-scale vascularized cardiac constructs fabricated using patient-derived cells and dECM bioinks, further validating the feasibility of personalized whole-organ bioprinting approaches [29].

The future integration of these bioprinted constructs with cutting-edge microfluidic perfusion bioreactors and dynamic conditioning systems could potentially address existing transplantation limitations by enabling the on-demand fabrication of patient-specific, fully functional cardiovascular tissues and organs. Nonetheless, considerable technical, regulatory, and translational challenges must be overcome before the clinical implementation of fully bioprinted cardiovascular organs can become a reality.

## 6. Conclusions and Future Perspectives

The field of cardiovascular tissue engineering has advanced significantly, owing to progress in 3D bioprinting and bioink technologies. However, fully replicating the complex hierarchical structure and function of the cardiovascular system remains challenging. Success in this endeavor requires the integration of multiple strategies, including precise cell patterning, robust vascularization, mechanical resilience, and tissue-specific maturation. The cardiovascular system exhibits a particularly strong interdependence between its structural configuration and functional performance, primarily because of its critical role in pumping blood and transporting essential nutrients, oxygen, and signaling molecules throughout the body. The complex geometry and hierarchical arrangement of myocardial fibers and vascular networks directly influence their mechanical efficiency, contractile performance, and hemodynamic stability. Consequently, precise bioprinting technologies that faithfully replicate the native structural architectures are indispensable for restoring or mimicking these essential physiological functions. Among the current bioprinting modalities, extrusion-based bioprinting (EBB) remains the most versatile platform for fabricating cell-laden multilayered cardiovascular constructs [45,122]. This status is largely due to the EBB’s capacity to process high-viscosity hydrogels and achieve spatially controlled deposition of multiple cell types. In particular, EBB is well suited for printing hydrogel-based bioinks reinforced with conductive or elastic additives, a strategy used to create cardiac patches and perfusable vascular tissues. Melt electrowriting (MEW) has emerged as a strategy complementary to EBB, enabling the fabrication of fibrous scaffolds with microscale resolution and well-defined architectures. These microfibrous frameworks enhance the mechanical stability and guide cell alignment, both of which are essential for mimicking the anisotropic structure of the myocardium and vascular walls [59,123]. Two-photon polymerization (2PP) offers another dimension of innovation by enabling submicron precision in the construction of microvascular networks and heart-on-a-chip platforms. Its ability to generate complex geometries with exceptionally high resolution makes 2PP ideal for fabricating capillary-scale guidance structures that influence endothelial cell behavior and perfusion capacity [115,124]. In addition, in situ bioprinting has gained traction as a clinically relevant approach to real-time tissue repair. This method allows surgeons to directly deposit bioinks into a defect site, conforming to patient-specific anatomy, and bypassing the limitations of ex vivo scaffold preparation. In cardiac applications, in situ bioprinting has demonstrated the potential for delivering personalized therapeutic patches after myocardial infarction, thereby promoting rapid integration with the host tissue [125]. The convergence of these technologies, EBB for bulk tissue fabrication, MEW and 2PP for microstructural fidelity, and in situ bioprinting for direct clinical applications, represents the most promising strategy for engineering functional cardiovascular tissues. Research efforts should prioritize the development of adaptive bioinks, multimaterial bioprinting systems, and real-time monitoring capabilities, as these advances will collectively enhance the functional maturation, vascular integration, and safety of bioprinted grafts. In parallel, the successful clinical translation of these innovations will require standardized protocols, scalable manufacturing platforms, and robust regulatory frameworks. In addition, reproducible large-scale manufacturing under GMP conditions, together with rigorous long-term preclinical validation, will be essential to ensure both safety and clinical translatability. Beyond the strategies currently in use, future innovations may arise from the integration of high-resolution microscale fabrication techniques with bioactive materials. Although methods such as 2PP and MEW have not yet been extensively applied to build large-scale functional cardiac tissues, their inherent capabilities offer unique advantages. Specifically, 2PP can serve as a cornerstone for constructing biomimetic capillary networks with precise luminal geometry and shear-guiding cues, whereas MEW can yield structurally reinforced scaffolds that facilitate long-term mechanical stability and promote cardiomyocyte alignment. Furthermore, combining MEW-patterned microfibers with embedded 2PP-fabricated capillary guidance structures could yield hierarchical anisotropic constructs that recapitulate both the electrical and vascular architectures of the native myocardium. Notably, this integrated approach is yet to be fully explored in the current literature. The potential of such an approach underscores the importance of bridging nanoscale precision with macroscale fabrication to overcome the persistent challenges associated with vascular integration and long-term construct maturation. Therefore, a forward-looking strategy should emphasize the co-development of bioactive stimuli-responsive bioinks with multimodal bioprinting systems. Such integration could facilitate the on-demand fabrication of personalized cardiovascular tissues with precise microvascular resolution, electromechanical synchrony, and mechanical resilience. Ultimately, the synergistic implementation of these advanced technologies signals a transformative era in personalized cardiovascular regeneration and precision biomedicine. Emerging approaches using artificial intelligence and machine learning allow for the predictive modeling of print parameters, crosslinking conditions, and rheological properties, accelerating the optimization of novel bioink formulations for cardiovascular applications.

## Figures and Tables

**Table 2:** Comparison of major bioprinting modalities for cardiovascular applications.

Method	Resolution	Cell Viability	Scalability	Material Compatibility	Cost	Clinical Readiness	Limitations	Reference
Extrusion	100–1200 µm	40–86% (variable due to shear stress), capable of high-cell density printing for thick tissues	Suitable for large construct fabrication (e.g., vascular grafts, heart tissue scaffolds), but slow for high-resolution complex structures	High-viscosity hydrogels (alginate, gelatin, PEG blends), cell-laden bioinks (fibroblasts, cardiomyocytes, stem cells), thermoplastics (PCL, PLA, PLGA). Specific cardiac bioinks like dECM, PEG, Laponite	Low-Medium	Widely used in preclinical studies; early clinical trials for cardiovascular constructs (e.g., valves, vascular grafts)	Shear stress reduces cell viability, low microscale resolution, structural fragility, and limited porosity	[18,39,41,43,45,48,50,62,68]
FRESH	Approx. 100 µm	High (due to cell-friendly environment of the support bath, beneficial for sensitive cardiac cells)	High potential for patient-specific, full-scale tissue production (e.g., heart models), though current software limitations exist	Low-viscosity and fragile ECM-based bioinks (collagen I, dECM, fibrin, Matrigel), cell-laden bioinks (fibroblasts, cardiomyocytes, stem cells); highly relevant for cardiac ECM mimicry	Medium	Preclinical demonstrations (e.g., patient-specific heart and valve models); no direct clinical translation yet	Post-processing complexity, support removal challenges for vascular structures, and software limitations for non-planar designs	[9,12,15,16,19,44,69,70,71,72,73,74,75,98,99]
DLP/SLA	10–150 µm	High	Faster than point-by-point methods, but scalability is limited for very large constructs; beneficial for intricate vascular networks	Photocurable hydrogels (PEGDA, GelMA, ECM-methacrylates); used for vascularized constructs	Medium-High	Strong preclinical successes in vascularized tissues (e.g., perfusable liver constructs, HUVEC-based anastomosis); still experimental with no clinical translation	Restricted to photocurable inks, phototoxicity risks for sensitive cells, and variable resolution between DLP and SLA	[16,83,84,85,86,87,88]
MEW	Fiber diameter: 2–50 µm	Not applicable	High scalability for structural scaffold fabrication, which can then be vascularized	Thermoplastics (PCL, PLA, PLGA); provides mechanical strength; can be integrated with hydrogels for enhanced bioactivity	Medium	Ongoing in vivo studies; high translation potential for structural applications (e.g., bone regeneration, cardiovascular grafts)	Thermal degradation, limited bioactivity requiring hybrid strategies, and fiber bridging from electrostatic attraction	[76,77,78,79,80,81,82]
2PP	Submicron (up to 100 nm possible, <500 nm for biodegradable materials).	Over 90% possible (improved with recent technological advancements)	Very low; excellent for micro-models and vascular topologies	PEGDA and other photocurable hybrid hydrogels, functionalized photopolymers, nanoparticle-loaded systems; used for vascular models	High (costly femtosecond lasers, low throughput)	Proof-of-concept stage; used for vascular models and complex biomimetic tissues (millimeter-scale)	Very low throughput, high cost, and difficulty in large-scale production of full-scale cardiovascular organs	[89,90]

## Abbreviations

CNT	Carbon nanotube
HCAECs	Human coronary artery endothelial cells
CMs	Cardiomyocytes
hMSCs	Human mesenchymal stem cells
GelMa	Methacrylated gelatin
HAGM	Hyaluronic acid glycidyl methacrylate
LAP	Lithium phenyl-2,4,6-trimethylbenzoylphosphinate
PEGDA	Poly(ethylene glycol) diacrylate
dECM	Decellularized extracellular matrix
hCPCs	Human cardiac progenitor cells
hiPSC-CMs	Human induced pluripotent stem cell-derived cardiomyocytes
hCFBs	Human cardiac fibroblasts
HA	Hyaluronic acid
HUVECs	Human umbilical vein endothelial cells
PCL	Polycaprolactone
TOCNF	TEMPO-oxidized cellulose nanofibrils (TOCNF) (where TEMPO: 2,2,6,6-Tetramethylpiperidine-1-oxyl)
FRESH	Freeform reversible embedding of suspended hydrogels
MeHA	Methacrylated Hyaluronic Acid
PCL	Poly ε-caprolactone
HUVSMCs	Human umbilical cord vein smooth muscle cells
pHMGCL	Poly(hydroxymethylglycolide-co-ε-caprolactone
ciPTECs	Conditionally immortalized proximal tubular epithelial cells
GM-HA	Glycidal methacrylate–hyaluronic acid
SCID	Severe combined immunodeficiency
hiPSC-HPCs	hiPSC-derived hepatic progenitor cells
GBM	Glioblastoma multiform
GSCs	Glioblastoma stem cells
NPCs	Neural progenitor cells
ASCs	Adipose-derived stem cells
nHA	Nanocrystalline hydroxyapatite
OMA	Oxidized methacrylic alginate
TAZ	4,4′-(1,2-ethenediyl)bis [3-sulfobenzenediazonium]dichloride
DAS	4,4′-(1,2-ethenediyl)bis [2-(3-sulfophenyl)diazenesulfonate]
